# Dynamics of the milk microbial community during subacute ruminal acidosis with or without intramammary lipopolysaccharide challenge in dairy cows

**DOI:** 10.1186/s42523-025-00499-5

**Published:** 2026-02-26

**Authors:** Viktoria Neubauer, Siska Aditya, Narciso M. Quijada, Stefanie Urimare Wetzels, Monika Dzieciol, Poulad Pourazad, Qendrim Zebeli, Evelyne Selberherr

**Affiliations:** 1 FFoQSI GmbH – Austrian Competence Centre for Feed and Food Quality, Safety, and Innovation, Tulln, 3430 Austria; 2 Centre for Food Science and Veterinary Public Health, Clinical Department for Farm Animals and Food System Science, University of Veterinary Medicine, Vienna, 1210 Austria; 3 Research Group of Feed and Food Safety, Research Center for Food Technology and Processing, National Agency for Research and Innovation, Yogyakarta, 55861 Indonesia; 4 Institute of Functional Biology and Genomics (IBFG), CSIC – University of Salamanca, Salamanca, 37008 Spain; 5 Veterinary Practice Veronika Brugger, Zell am See, 5700 Austria; 6 Cargill Animal Nutrition, Cargill Incorporated, Wayzata, 55391 MN USA; 7 Centre for Animal Nutrition and Welfare, Clinical Department for Farm Animals and Food System Science, University of Veterinary Medicine, Vienna, 1210 Austria; 8 Christian Doppler Laboratory for Innovative Gut Health Concept of Livestock, Vienna, 1210 Austria

**Keywords:** Udder health, High-grain feeding, Mastitis, Somatic cell count, Milk amyloid A, Gut-mammary gland axis

## Abstract

**Background:**

Lipopolysaccharides (LPS) from pathogenic Gram-negative bacteria play a key role in the pathophysiology of mastitis. Subacute ruminal acidosis (SARA) induces rumen dysbiosis, leading to LPS translocation and systemic immune activation. This study investigated the effects of a high-grain diet and intramammary LPS challenge on the milk microbiome of dairy cows. Cows were first fed a baseline control diet (day-7 to d-1; CON; 40% grain; *n* = 18). On d1, 12 cows were switched to a SARA diet (60% grain). On d30, six SARA (SARA_LPS) and the six CON cows (CON_LPS) were challenged intramammarily with LPS, while the other six SARA cows received a placebo (SARA_PLA). No CON_PLA group was enrolled. Milk samples were collected on d-2 (before feeding challenge), d30 (after feeding challenge; before LPS), and d32 (after LPS), and analysed using 16S rRNA gene amplicon sequencing and qPCR.

**Results:**

During the feeding phases, more genera (70.1%) increased in CON than SARA cows, whereas more (65.3%) genera decreased their relative abundance in SARA compared to CON (*p* < 0.001). This decline persisted in SARA_PLA cows, with more genera (62.4%) decreasing their abundance (*p* < 0.001). However, LPS injection reversed the trend of the feeding effect, with more genera (79.3%) increasing in SARA_LPS cows in comparison to the other two groups, while more genera (85.5%) decreased in CON_LPS (*p* < 0.001) in comparison to the other two groups. Alpha diversity correlated positively with bacterial cell equivalents. Of all genera, 22.1% correlated negatively with milk amyloid A (MAA), which increased post-LPS injection, 21.7% positively with lactose, and 13.4% positively with milk urea. SCC showed significant differences in beta-diversity, but no distinct visual clustering nor many correlations.

**Conclusion:**

The microbial dynamics suggest that high-grain diet and the LPS injection influence the milk bacterial community. More taxa correlated with MAA than with SCC, suggesting that MAA may better reflect immune-microbial interactions in milk. A roughage-rich diet promoted higher microbial abundance, whereas high-grain feeding reduced abundance over the timespan of 30 days. Intramammary LPS challenge decreased absolute abundance in CON but increased it in SARA cows, suggesting a diet-dependent immune modulation of the mammary environment. These findings indicate that mammary gland epithelial integrity and immune mediators jointly shape the milk microbiome under metabolic and inflammatory stress.

**Supplementary Information:**

The online version contains supplementary material available at 10.1186/s42523-025-00499-5.

## Background

In the past, it was believed that the milk of an udder-healthy cow is a sterile fluid and would only harbour pathogens during mastitis. The use of culture-independent methods has prompted a re-evaluation of this dogma, revealing that microbes are also present in a healthy udder [1]. Although there is a large discrepancy between results coming from culture-dependent and -independent methods, it is accepted that at least the DNA or immune cell-processed parts of living microbes are found in the milk and the udder [2–5]. Moreover, in infectious mastitis cases with negative culture results, we need to consider (yet) non-culturable microbes [6]. Furthermore, not only the presence of viable bacteria, but also their antigens can activate the local udder-immune response [7]. Therefore, understanding the microbial community of the udder and milk, even when transient, is crucial for comprehending infectious mastitis and udder health.

There are two main ways for microbes to enter the udder, either endogenously, via a systemic route, such as the gut-mammary gland axis [8, 9], or from the environment, including bedding, milking equipment, or the oral cavity of the calve, via the teat canal or udder-injuries [10, 11]. Thus, the diet and intestinal health of the cow may influence the milk microbiome. Microbiota, or their metabolites, may be endogenously translocated from the gut to the udder [12, 13], or changes in the faecal microbes, which are highly dependent on diet [14], might influence the environmental presence of microbes [15].

Dairy cattle are commonly fed high-grain diets to meet the high energy demands for milk production. However, this feeding practice often leads to subacute ruminal acidosis (SARA), which not only alters microbial composition throughout the digestive tract [16] but also triggers an excessive release and translocation of lipopolysaccharides (LPS) into the rumen fluid [17] and the hindgut [18]. This highly proinflammatory agent, derived from the outer leaflet of the membrane of Gram-negative bacteria (GNB), along with translocated microbes, may enter the systemic circulation and potentially influence the local microbiome or immunity in various internal organs [19].

To date, only a few studies have investigated whether dietary changes influence the cows’ milk microbial community [20–22]. However, none of these studies evaluated effects over time within the same animals. Therefore, this study aimed to examine the impact of a high-grain diet, with its resulting ruminal dysbiosis as well as systemic effects, on the milk microbiome during a SARA feeding model. Our respective hypothesis was that cows experiencing SARA would have a decreased abundance of the milk bacterial community over time, compared to cows on a moderate-grain diet.

When cows experience mastitis, the microbial diversity in the milk decreases, while certain genera become highly abundant [23, 24]. Additionally, the production of microbial compounds that can act as toxins for the host, such as LPS, elicits a strong local to systemic immune response [25]. This immune response is directed at eliminating pathogens and may simultaneously influence the milk microbiome [26]. To date, no study has specifically investigated the impact of the immune response on the milk microbiome in the absence of a pathogenic challenge in dairy cows during moderate and high-grain feeding. Therefore, the second aim of this study was to induce a strong immune stimulation through intramammary LPS injection to assess potential immune-driven changes in the milk microbial community. We used an experimental model that allowed us to evaluate the individual and the combined effects of the LPS-induced mammary inflammation during a moderate-grain or high-grain SARA-inducing diet. Our second hypothesis was that LPS injection would influence the abundance of the milk microbiome differently, depending on whether the cows already experienced SARA or not.

It is well known from mastitis infections that, depending on the severity of infection, the histo-anatomical separation of the udder quarters prevents pathogen spread between them [10]. This is one reason why previous work has shown a different microbial composition between healthy quarters and those developing signs of mastitis [6]. However, to date, no study has investigated the physiological microbial community differences between two quarters of the same cow that shows no signs of infection. Our approach provided a first insight into the physiological composition of the individual quarter microbial community, under the same endogenous or exogenous conditions in a single cow. Thus, the third aim of this study was to compare the microbial community composition of two healthy quarters at the same sampling timepoint of all cows enrolled in this study. Our third hypothesis was that two quarters of the same cow would harbour similar microbial communities.

## Materials and methods

This study was part of a larger project. The effects of the high-grain diet and an intramammary LPS injection on several rumen, blood, milk, fecal, and health parameters have been published in our accompanying papers [17, 27–29]. The purpose of the current project was to elaborate the effects of the diet and the LPS challenge on the milk microbial community.

The experimental procedures were approved by the Institutional Ethics Committee of the University of Veterinary Medicine Vienna (Vetmeduni Vienna) and the National Authority of Austria according to §26 of the Law for Animal Experiments, Tierversuchsgesetz 2012- TVG (GZ BMWFW-68.205/0096-WF/V/3b/2015).

### Feeding groups and intramammary LPS infusion

The feeding trial with cows, groups, diets, and SARA induction protocol has been described in detail by Aditya et al. [29]. In brief, all 18 Simmental milking cows (eight primiparous, ten multiparous; 3 ± 1.8 lactations; 66 ± 20.5 DIM) enrolled in the trial received a moderate-grain diet (40% grain on dry matter (DM) basis) as Baseline diet from seven days (d-7) to one day (d-1) before the challenge. From day 1 (d1) on, six of the cows continued to be fed the moderate-grain diet throughout the trial phase (d1 to d32) and were thus considered as the control group (CON). The remaining 12 cows underwent an intermittent SARA challenge, receiving 60% grain (DM basis) from d1 to d8 and d16 to d32, intermitted by a moderate-grain diet phase from d9 to d15, and thus considered as SARA group. SARA induction was confirmed by pH < 6.0 for more than 314 minutes per day, while the CON group stayed below this threshold (*P*  < 0.01), as published by our accompanying paper [27].

The intramammary LPS infusion was performed on d30 of the experiment, 3 h after the morning milking. Half of the SARA cows (SARA_LPS; *n* = 6) and all CON cows (CON_LPS; *n* = 6) received a direct injection of 50 μg LPS from *Escherichia coli* (O26:B6; Sigma-Aldrich Inc., St. Louis, MO), diluted in 10 mL of sterile NaCl solution (B. Braun, Melsungen, Germany) into the left front quarter using a sterile plastic syringe (BOVIVET Patteansats, Langeskov, Denmark). The remaining six SARA cows (SARA_PLA) received an injection of 10 mL sterile NaCl solution as a placebo [29]. The experimental trial is illustrated in Figure S1, Additional file 1. Due to the limitations of conducting an extensive large-animal trial, no CON_PLA group was included in this study.

### Milk sampling

Milk samples for DNA extraction and content analysis were collected from all cows right before the morning milking on d-2 (Baseline, all cows on 40% grain), d30 (just before the injection of LPS or PLA), and d32 (45 h after the injection of LPS or PLA) from the left front quarter. On d30, additional samples from the right front quarter were taken for community comparisons between quarters of each cow (Figure S1, Additional file 1). After cleaning the teats with a commercial teat cleaner and drying them with disposable paper towels, each teat end was rubbed with a disposable towel saturated in 70% ethanol. The first streams of milk were discarded and then two 50 mL vials were filled with approximately 40 mL of milk each. Samples for microbial community analysis and milk amyloid A (MAA) were put at −80 °C until further processing. The concentration of MAA was analyzed using a commercial kit specific for milk (Tp-807; Tridelta Development Ltd, Maynooth, Ireland). Samples for milk contents were preserved with Bronysolve (ANA.LI.TIK, Wien, Austria) and stored at 4 °C until analysis for SCC, protein, fat, lactose, urea, pH, and total solid-non-fat (SNF) using Combifoss (Foss, Hillerød, Denmark). Gloves were changed between cows.

### DNA extraction

The milk samples were thawed on ice, and sample preparation was performed following the Matrix-Lysis protocols, as previously described [30–32]. All reagents for the Matrix-Lysis protocol were purchased from Merck (Germany), except for SDS, Tricine (Sigma-Aldrich, Germany), and Lutensol AO-07 (BASF, Netherlands). Briefly, 12 mL of the milk sample, in duplicate, was mixed with 33 mL lysis buffer (2 M MgCl_2_, 50 mM Tricine pH 7.6; 1% Lutensol AO-07). The samples were incubated horizontally in a SW23 shaking water bath (Julabo, Germany) at 37 °C and shaken at 200 rpm for 30 min. After incubation, the samples were centrifuged at 3,220 × g for 30 min at room temperature. The supernatant was discarded, and the pellet obtained from two falcon tubes was pooled. Subsequently, the pellet was resuspended in 40 mL washing buffer (0.35% Lutensol AO-07, 1 × PBS) and incubated horizontally at 37 °C in a water bath at 200 rpm for 30 min. The sample was centrifuged at 3,220 × g for 30 min, and the supernatant was gently discarded.

The remaining bacterial pellet was resuspended in 500 µL of sterile 1 × PBS and washed twice in 1.3 mL of 1 × PBS, with an additional centrifugation step for 8 min at 4,000 × rpm. The complete pellet obtained from the last step of the Matrix-Lysis protocol was then subjected to DNA extraction using the NucleoSpin®Tissue kit with the support protocol for Gram-positive bacteria (GPB), according to the manufacturer’s instructions (Macherey-Nagel, Germany). Briefly, the pellet was incubated in 180 μL lysis buffer (20 mM Tris/HCl; 2 mM EDTA; 1% Triton X-100; pH: 8) supplemented with lysozyme (20 mg/mL; Sigma-Aldrich, Austria) with constant shaking (1,000 × rpm) for 1 h at 37 °C and incubated with Proteinase K (25 μL, 20 mg/mL) overnight at 56 °C. The DNA was eluted twice with 15 µL (2 × 15 µL) of 70 °C prewarmed diethylpyrocarbonate (DEPC)-treated water, instead of using the C6 solution. For each cow, one DNA extraction negative control was included (NK; *n* = 18).

DNA concentration was determined using the Qubit 2.0 Fluorimeter and the high-sensitivity and broad-range dsDNA Assay Kit (ThermoFisher Scientific, Austria). To verify DNA content and potential contaminations, the DNA samples, processing, and extraction negative controls were analysed using 16S rRNA gene-targeted PCR (27F/1492 R). A standard 16S rRNA gene PCR (initial denaturation at 95 °C for 5 min, then denaturation at 94 °C for 40 s, annealing at 52 °C for 40 s and elongation at 72 °C for 1 min, 35 cycles, final elongation at 72 °C for 7 min) was used with the primers 27F (5′-AGAGTTTGATCMTGGCTCAG-3′, positions 8 to 27 in *E. coli* 16S rRNA coordinates) and 1492 R (5′-GGYTACCTTGTTACGACT *T*-3′, positions 1492 to 1510 in *E. coli* 16S rRNA coordinates) [33]. The amplified products were visualized using the GelDoc Go (Bio-Rad, Austria) after electrophoresis in a 1.5% TAE-agarose gel.

### Quantitative PCR (qPCR)

DNA (1 µL) from all milk samples was pooled, and a conventional PCR was performed using the primer set 341F (5′-CCT ACG GGA GGC AGC AG-3′) and 534 R (5′-ATT ACC GCG GCT GCT GG-3′) [34] to generate the required amount of DNA for the qPCR standard. The resulting DNA was purified using the innuPREP PCRpure Kit (ist Innuscreen GmbH, Berlin, Germany) and quantified with the Qubit® ds BR Assay Kit (Thermo Fisher Scientific, Austria). The purified standard was 10-fold diluted and tested in duplicate to determine the copy numbers of bacterial 16S rRNA genes.

The DNA samples were assayed in duplicate in a 23 μL reaction mixture, which contained 12.7 μL DEPC-treated water, 2.5 μL 10 × buffer, 1.0 μL 2 mM MgCl_2_ (stock concentration 50 mM), 2.5 μL of each primer (stock concentration 2.5 μM), 0.5 μL of EvaGreen fluorescent DNA stain (JenaBioscience, Jena, Germany), 1.0 μL of dNTP Mix (stock concentration 20 mM, 5 mM of each dNTP; Thermofisher, Vienna, Austria), 0.3 μL of Platinum Taq DNA polymerase (5 U/μl; Thermo Fisher Scientific, Vienna, Austria), and 2 μL of the diluted template (gDNA). The quantification of DNA was performed in an Mx3000P qPCR instrument (v.4.10; Stratagene, La Jolla, CA, United States) with an initial denaturation at 95 °C for 3 min, followed by 45 cycles of 95 °C for 5 s, 60 °C for 20 s. To determine the specificity of the amplifications, dissociation curves after each reaction were recorded and carried out at 95 °C for 1 min, followed by complete annealing at 50 °C for 30 s, and a gradually increasing temperature up to 95 °C. Post-run melting curves were checked for the presence of multiple peaks due to primer-dimers or non-specific amplification. Additionally, negative controls without templates were included in each qPCR run.

The final copy numbers of total bacteria were calculated by multiplying the mean of the copy number (bacterial cell equivalents, BCE) per sample, as determined by qPCR. The BCE of the 16S rRNA gene was determined by assuming that based on the entered amount of DNA (1.0 ng) weight and 194 bp length of the template, 1.0 ng of DNA equals 4.78E + 9 number of copies (dsDNA copy number calculator). Additionally, the median of six 16S rRNA gene copy numbers in bacteria (derived from 40,019 genomes [35], was used to extrapolate the final copy numbers.

### Sequencing

The extracted DNA from the 90 samples (72 milk and 18 NK) was sent for Illumina MiSeq Amplicon Sequencing (Microsynth, Balgach, Switzerland) to target the V3-4 regions of the 16S rRNA gene, using the forward 341F_ill (CCTACGGGNGGCWGCAG) and reverse 802R_ill (GACTACHVGGGTATCTAATCC) primers, producing an approximate amplicon length of 460 bp. Library preparation, quality control, and sequencing were performed by Microsynth (Balgach, Switzerland) yielding a median of 57,882 raw paired-end sequences per sample (range 12,475–216,362).

### Sequence data processing

The quality of the raw paired-end reads was first assessed by using FastQC [36] and MultiQC [37], while potential residual barcodes and adapters were removed by using Trimmomatic [38]. The remaining sequencing reads were further processed using QIIME2 v.2.2023.9 [39] as follows: Quality filtering of the reads, merging of the paired ends, chimera removal, and identification of Amplicon Sequence Variants (ASVs) was performed by using the q2-dada2 plugin [40], with a maximum number of expected errors of 2 and truncating the reads (by enabling –p-trunc-len option) to 247 and 240bp for forward and reverse reads, respectively. A total of 142 potentially contaminant ASVs were removed by using decontamR [41] (enabling *isContaminant* algorithm, prevalence method, threshold = 0.5), accounting the information provided by the 18 extraction negative controls.

Taxonomy was assigned to the ASVs by using a pre-trained Naïve Bayes classifier based on SILVA SSU v138.1 NR99 [42], with the q2-feature-classifier plugin [43]. All ASVs assigned to “mitochondria”, “chloroplast”, “eukaryotae”, and “unassigned” were excluded by using the taxa plugin and the filter-table option, resulting in a total of 8,321 ASVs remaining from contamination QC. ASV abundance based on sequence counts per ASV was normalized to genome copy number (GCN) using the *g-norm* plugin [44] with the *copy-num-normalize* option and the information contained in rrnDB [35]. After ASV QC and GCN, all ASVs with low prevalence (i.e. present in only one sample) or low abundance (i.e. less than 10 GCN counts in total) were removed from the dataset. This resulted in a total of 2,051 ASVs, with 715,098 GCN-sequences (mean 9,932 counts per sample, range 1,514 - 25,814) that were used for downstream analyses. The relative abundance of each ASV was multiplied by the BCE of the corresponding sample to calculate the absolute abundance of each ASV in each sample. For further statistical analysis and interpretation a phylum and a genus table were created, consisting of 15 different phyla and 277 different genera. Gram characteristics of the assigned genera was assigned manually using online sources [45–48].

A phylogenetic tree was generated using the q2-alignment [49] and q2-phylogeny [50] plugins. After GCN, alpha-and beta-diversity analyses were performed using the q2-diversity [51] and q2-taxa [52] plugins. The alpha diversity matrices Chao1, Shannon, Simpson (reciprocal Simpson 1/*D*), Simpson evenness, Dominance Index, Observed Features, and Singles were calculated to assess the diversity and distribution of taxa in the dataset. Alpha rarefaction curves and Good’s coverage were inspected to ensure that the microbial diversity was sufficiently covered at different rarefaction thresholds. For beta-diversity analyses, the dataset was rarefied to 4,296 GCN-sequences per sample, respectively, which excluded three samples for this comparison (cow 8 at d-2 and d30 (CON, LPS) and cow 12 at d-2 (SARA, PLA). All samples included in the beta-diversity analysis had a Good’s-coverage of > 94% at the selected threshold. Bray–Curtis dissimilarity and Weighted UniFrac distance matrices were computed.

### Statistical analyses

The milk contents, alpha-diversity parameters, phyla, and genera were not normally distributed among the independent variables but had homogenic variances. Therefore, non-parametric tests were used for all statistical approaches using SPSS (IBM SPSS Statistics 28.0.0.0).

In the sample from cow1 at d30 from the right front quarter (SARA group), the relative abundances of the ASVs were inversely high in comparison to all other samples, the qPCR data was 73 times higher than the mean of all other samples, and it clustered significantly apart from the left counterpart in beta-diversity analysis. This indicated this sample as an outlier, leading to its removal from all further analyses concerning taxonomic differences. However, this sample would have only been relevant for comparing the right and left quarter of the cow. Consequently, the accompanying left quarter of cow 1 on d30 was also removed for this analysis, resulting in *n* = 17 cows for the comparison of the left and right quarter. The differences in alpha diversity, phyla, and genera between the two quarters were assessed with the Wilcoxon test for associated samples, with the individual cow as the connecting factor. Exact one-way significance was calculated. To rule out a stronger influence of quarter compared to the feeding group or treatment group, a regression analysis with backward elimination was performed for alpha diversity parameters and phyla. Quarter was consistently the first variable to be eliminated from the model (*p* > 0.8), therefore we did not assume a greater impact of quarter than of the feeding or treatment groups.

The aim of this study was to elaborate the effects of diet and treatment over time in the same animal. To analyse the effect of the feeding groups (CON, SARA) on milk content, MAA, alpha diversity, and the single phyla and genera, the values for the cows on d-2 (Baseline) and d30 (CON, SARA feeding groups) were considered as paired samples, with cow as pairing variable, and analysed using the Wilcoxon Test. Exact one-way significance was calculated.

To analyse the effect of the LPS injection on milk content, MAA, alpha diversity, and the single phyla and genera, the values for the cows on d30 (4 h before injection) and d32 (43 h after injection) were considered as paired samples, with cow as pairing variable, and SARA_PLA, SARA_LPS, and CON_LPS as grouping variable, and analysed using Wilcoxon Test. Exact one-way significance was calculated. An absolute abundance table for phyla and genera in the feeding and treatment groups is given in Additional file 2.

For interpretation and graphical presentation, the fold change between the days was calculated for each feeding group (CON, SARA: d30/d-2) and treatment group (SARA_PLA, CON_LPS, SARA_LPS: d32/d30) for milk contents, phyla, and genera. Chi-square tests were applied to examine the overall directional trends (decrease or increase) of phyla, genera, and the corresponding Gram characteristics in response to the feeding and treatment over time. Bot inter- and intra-group differences were calculated with two sided pairwise comparisons between the groups using Bonferroni correction.

Beta-diversity inter-sample differences were analysed using PERMANOVA in QIIME2 using the beta-group-significance method, which calls the adonis function from the R package vegan. Each analysis included a single fixed factor (Feeding group, LPS group, SCC group) as the explanatory variable. No random effects or blocking structures were included. Significance was assessed using q-values (FDR-corrected P-values).

Correlation analyses were performed using the Spearman rank correlation factor (*r*) between BCE, milk content, MAA, alpha diversity, phyla, and genera. Correlations with *P* ≤ 0.05 were interpreted as poor with *r* |< 0.3|, fair with *r* |≥0.3 – < 0.5|, moderate with *r* |≥0.5 – < 0.7|, strong with *r* |≥0.7 – < 0.9|, and substantial with *r* |≥0.9–1.0|. For all approaches, differences between groups were considered as significant when *P* ≤ 0.05 or as tendency when 0.05 < *P* ≤ 0.1.

## Results

This study analysed the effect of a high-grain diet (60% grain on a dry matter basis), inducing subacute ruminal acidosis (SARA), compared to a moderate-grain control diet (CON, 40% grain on a dry matter basis), as well as the additional effect of intramammary LPS injection (SARA_LPS, CON_LPS) compared to a placebo (SARA_PLA) on the milk microbial community over time. Milk parameters were investigated using correlation analysis to explore potential relationships with the genera. Furthermore, this study investigated how the microbial community of two quarters of the same cow differs at the same sampling timepoint.

### Milk constitutes changes during SARA and LPS challenge

While the effect of the experimental model on the collective milk samples was published before [17, 29], the milk parameters in this study were measured in the quarter milk samples which were further subjected to DNA extraction and sequencing. During the feeding trial, the milk constitutes showed significant, but numerically small changes for both CON and SARA cows, but SCC, MAA and bacterial measures did not change significantly (Table 1). After LPS infusion, the DNA amount, SCC, and MAA increased significantly or tendential in both CON_LPS and SARA_LPS cows, with BCE additionally increasing in SARA_LPS but not CON_LPS, while values remained stable in SARA_PLA cows (Table 1). The SARA_PLA group had a numerically large fold change (FC) for SCC, however not significant, as this effect was mainly attributed to one outlier sample, which showed an increase in SCC from d30 to d32 in this group. Absolute values for milk constitute are given in Additional file 1, Table S1.

**Table 1:** Parameters measured in the milk samples during moderate-or high-grain feeding and LPS challenge

Milk parameter	FC d-2 to d30		FC before to after injection		
	CON	SARA	SARA_PLA	CON_LPS	SARA_LPS
BCE	2.3	0.8	0.7	0.6	3.7**
DNA	1.2	1.9	1.1	3.7**	2.5**
SCC	1.2	0.2	139.8	231.3**	35.3*
MAA	0.4	2.2	1.2	3952.2**	212.2**
Fat	0.7**	0.5**	1.2**	1.4*	3.0*
Protein	1.0	1.0*	1.0	1.0	1.1
TSNF	1.0**	1.0	1.0	1.0**	1.1
Urea	0.7**	1.2	1.1	0.9	0.9
Lactose	1.0**	0.9**	1.0	1.0**	1.1*
pH	1.0*	1.0**	1.0	1.0*	1.0

Results are shown as fold change (FC) from d-2 (Baseline, all cows CON diet, 40% grain) to d30 in the CON group (*n* = 6) and the high-grain diet group (SARA, 60% grain; *n* = 12), or from d30, before intramammary treatment, to d32, 45 h after lipopolysaccharide (LPS; *n* = 12) or NaCl (Placebo, PLA *n* = 6) injection. BCE = bacterial cell equivalent; total DNA measured by Qubit; SCC = somatic cell count; MAA = milk amyloid A; TSNF = total solids non-fat; **P ≤ 0.05; *0.05 < *P* ≤ 0.1

### Alfa- and beta-diversity dynamics across con to SARA and the LPS challenge

There were no significant differences in the microbial alpha-diversity parameters during the feeding experiment between CON and SARA cows. Similarly, intramammary treatment with LPS or PLA did not affect alpha diversity (Table S2, Additional file 1).

Beta diversity was assessed using Bray Curtis dissimilarity and Weighted Unifrac distance matrices which represented 68.41% and 84.24% of the variation, respectively. Results were represented as Principal Coordinates Analysis (PCoA), which did not reveal any visual clustering based on changes in diet from CON to SARA, the intramammary LPS infusion, or SCC of the samples (Figure S2, Additional file 1). The PERMANOVA analysis showed a significant difference between the feeding groups in Bray-Curtis dissimilarity (*q* = 0.035) but only a tendency for Weighted Unifrac (*q* = 0.068), a significant difference for SCC classes (</ > 200,000 cells/mL) in Weighted UniFrac (*q* = 0.027) but only a tendency for Bray-Curtis (*q* = 0.057), and no effects of LPS treatment (*q* > 0.1).

### Microbial community changes during SARA and the LPS challenge

The samples were analysed as associated samples and grouped according to our main hypotheses: i) to study the different effects of a moderate-or high-grain feeding, the absolute bacterial abundances for CON (*n* = 6) and SARA (*n* = 12) groups at d30 were compared against the Baseline d-2, displayed as FC between the two timepoints. ii) to evaluate the effect of the intramammary LPS challenge, the absolute abundances for SARA_PLA, SARA_LPS, and CON_LPS were compared between d30 (just before injection), and d32 (approximately 45 h after injection), displayed as FC.

**Fig. 1:**
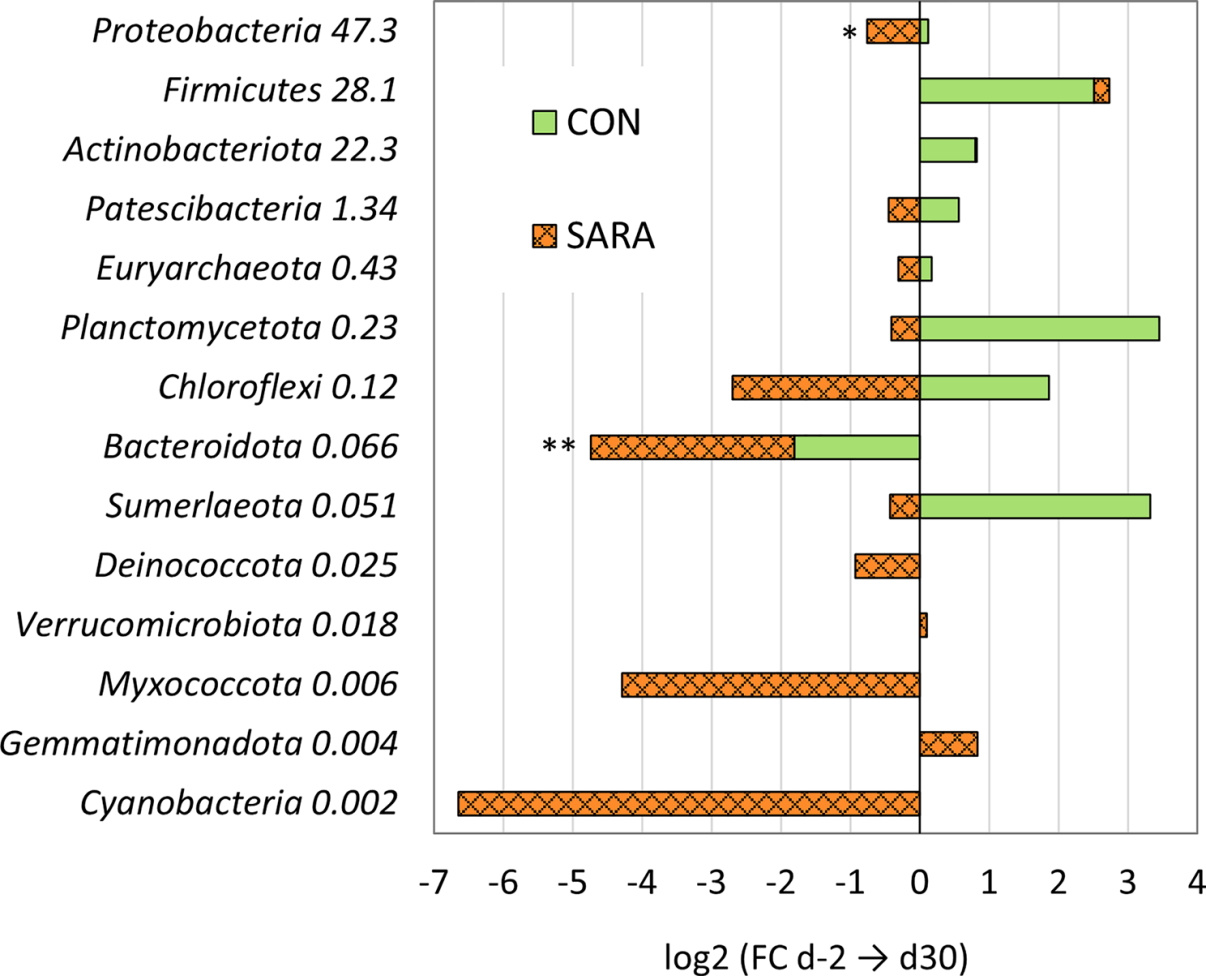
Dynamics of the phyla with the feeding model from day −2 to day 30. Shown as log2 fold change of absolute abundance of phyla from d-2 (Baseline, 40% grain) to d30 (CON, 40% grain, or SARA, 60% grain) of the experiment. The number next to the phylum names represents the overall frequency (%). **P ≤ 0.05; *0.05 < *P* ≤ 0.1

**Fig. 2:**
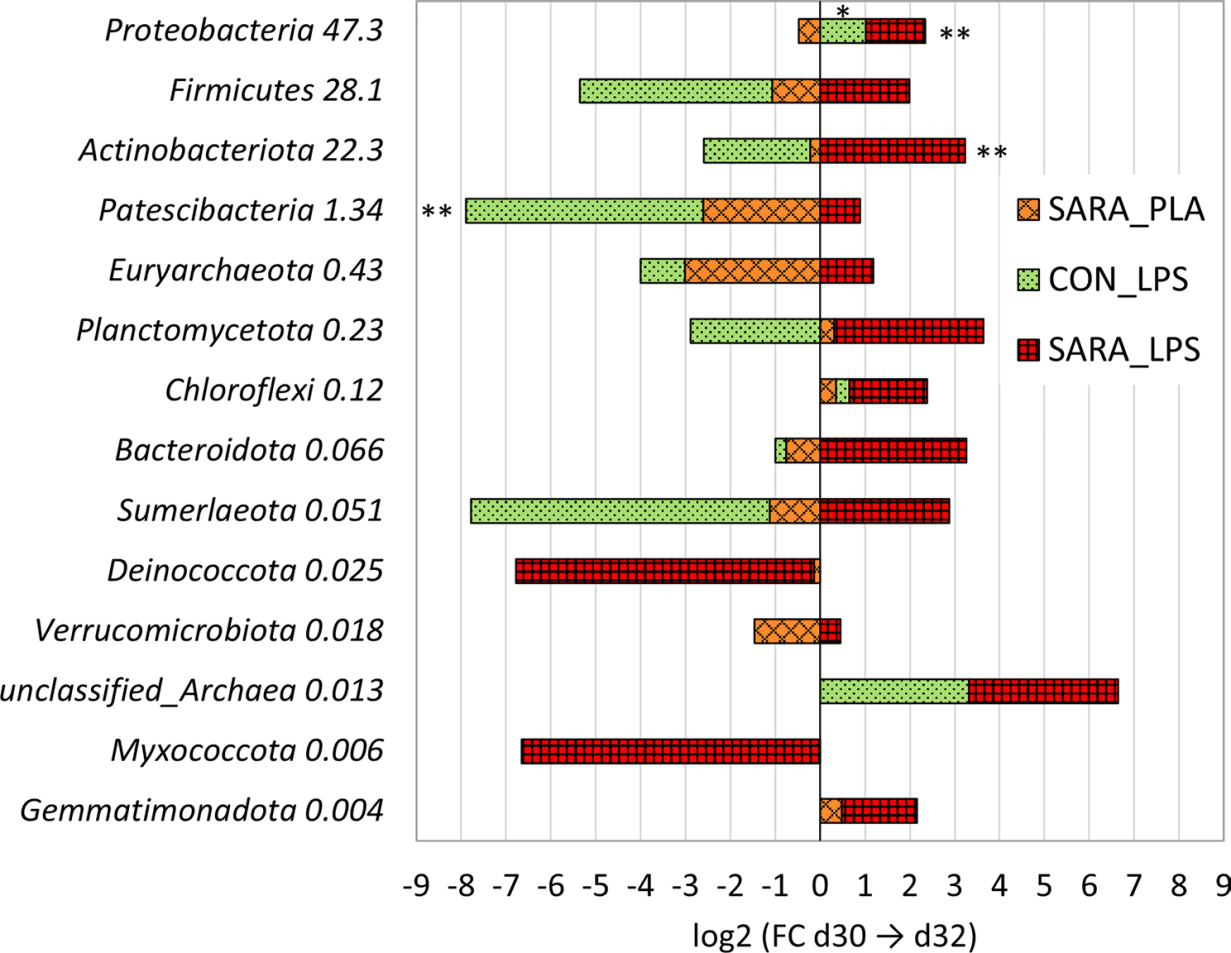
Dynamics of the phyla during LPS challenge compared to the placebo group. Shown as log2 fold change of absolute abundance of phyla from d30 (before injection) to d32 (45 h after injection of lipopolysaccharides (LPS) or placebo (PLA)) to cows fed 60% grain (SARA) or 40% grain (CON) of the experiment. The number next to the phlum name represents the overall frequency (%). **P ≤ 0.05; *0.05 < *P* ≤ 0.1

At phylum level, more (88.9%) phyla present in the CON samples increased their abundance from d-2 to d30 in contrast to SARA, while more (71.4%) phyla present in the SARA samples decreased during the trial in contrast to CON (χ^2^ (1) = 7.99, *P* = 0.005, Fishers Exact Test *P* = 0.007). Of those, *Proteobacteria* decreased significantly, and *Bacteroidota* tendential in the SARA group from d-2 to d30 (Fig. 1). After the injection of the placebo, the directional changes of the phyla in the SARA_PLA group persisted, with 75% of the phyla continuing to decrease their abundance, with no significant changes for single phyla. However, the LPS injection inversed the effects of the diet in the SARA_LPS group, with 85.7% of the phyla increasing their abundance, which was more than in SARA_PLA or CON_LPS (χ^2^ (2) = 11,74, *P*  = 0.003). Within those, the two highly abundant *Proteobacteria* and *Actinobacteriota* increased significantly from before to after LPS injection. In contrast, in the CON_LPS group, 70% of the phyla showed now a decrease after the LPS injection (χ^2^ (2) = 11,74, *P*  = 0.003), with a significant reduction observed in *Patescibacteria* and a tendential increase in *Proteobacteria* from before to after LPS injection (Fig. 2).

**Fig. 3:**
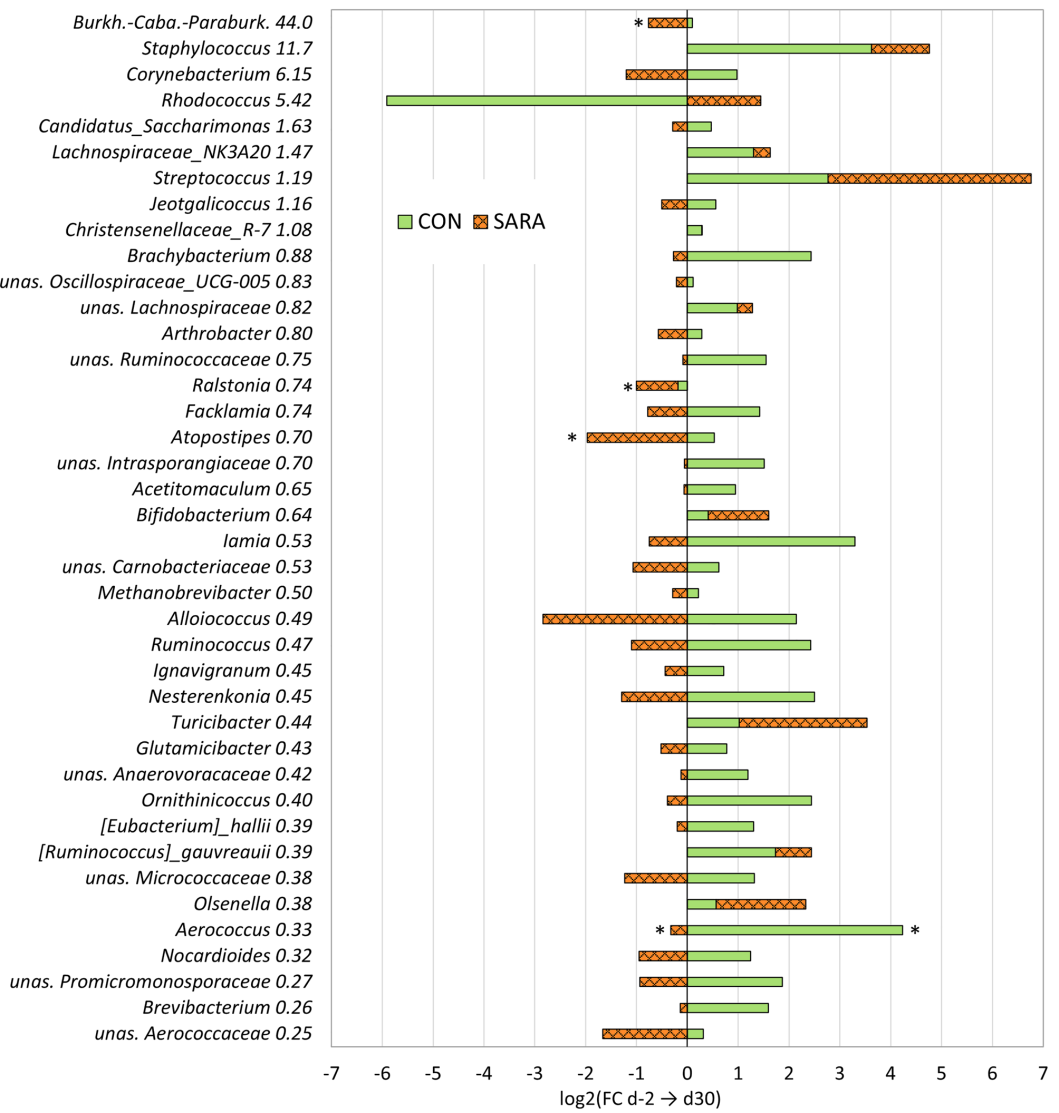
Dynamics of the 40 most abundant genera during CON and SARA feeding. The bars display the log2 of the fold change (FC) of the absolute abundance from the Baseline (d-2, all 40% grain) to the end of the feeding trial (d30). The CON cows received continuously a 40% grain diet and the SARA cows were challenged with an intermittent 60% grain feeding regime. The genera are sorted from high to low overall frequency, with the % reported next to the taxonomic name. unas. = unassigned; Burkh.-Caba.-Paraburk. = *Burkholderia-Caballeronia-Paraburkholderia*; unas. *Anaerovoracaceae* = *Anaerovoracaceae*_Family_XIII_AD3011; **P ≤ 0.05; *P ≤ 0.1

Among the total number of genera, more (70.1%) increased their absolute abundance in the CON cows during the experiment from d-2 to d30 in comparison with SARA cows (*p* < 0.001). Within those, half (51.5%) increased by at least 2-fold. During the intermittent 60% grain feeding, more genera (65.3%) decreased in the SARA cows in comparison to the CON cows (*P* < 0.001) (Table 2). Within those, 40.2% decreased by at least 2-fold (Fig. 3). The intra group Chi^2^ test also revealed that the number of increasing genera is higher within the CON group, and the number of decreasing genera is higher within the SARA group (Table 2). The injection of LPS on d30 caused an inverse effect on these directional changes, with more genera (85.5%) decreasing within the 45 h after injection in the CON_LPS group, while in SARA_LPS group then 79.3% increased (*P*  < 0.001) (Table 2). The SARA_PLA continued to decrease over time with 62.3% genera showing a decline (*P*  < 0.001), but not as strong as CON_LPS. Intra-group Chi^2^ test also showed that the number of genera increasing and decreasing was significantly different within the groups (*P*  < 0.001) (Table 2).

**Table 2:** Dynamics of the genera during the feeding model and intramammary challenge

**a) Feeding group**	**CON**	**SARA**		**Inter-group statistics**
	% (n)	% (n)		
**FC > 1** from d-2 to d30	70.13 (162)	34.69 (94)		χ^2^ (1) = 62.69, *P* < 0.001
**FC < 1** from d-2 to d30	29.87 (69)	65.31 (177)		
Intra-group statistics	χ^2^ (1) = 37.44, *P* < 0.001	χ^2^ (1) = 25.42, *P* < 0.001		
**b) Treatment group**	**CON_LPS**	**SARA_LPS**	**SARA_PLA**	
	% (n)	% (n)	% (n)	
**FC > 1** from d30 to d32	14.49_c_ (31)	79.34_a_ (192)	37.65_b_ (93)	χ^2^ (2) = 201.23, *P* < 0.001
**FC < 1** from d30 to d32	85.51_a_ (183)	20.66_c_ (50)	62.35_b_ (154)	
Intra-group statistics	χ^2^ (1) = 107.96, *P* < 0.001	χ^2^ (1) = 83.32, *P* < 0.001	χ^2^ (1) = 15.007, *P* < 0.001	

Number and proportion of the genera increasing (fold change (FC) > 1) or decreasing (FC < 1) with a) the feeding model from the Baseline (day −2, 40% grain) to d30 in CON cows, which received continuously a 40% grain, and SARA cows, which were challenged with an intermittent 60% grain feeding regimen; and with b) the treatment groups from before (d30) to after (d32) LPS injection in CON (40% grain) and SARA cows (60% grain) or placebo (PLA) injection in SARA cows. Inter- and intra- group differences were analysed using Chi^2^ tests and pairwise comparisons.

^abc^Denote differences among the same row at *P* < 0.001

Among all genera, 37.0% showed the same directional trends in both the CON and SARA cows, of which 54.5% increased and 45.5% decreased from d-2 to d30 in both groups. In contrast, 63.0% of the genera showed different dynamics over time for CON and SARA cows, with 62.2% increasing, 13.4% decreasing, and 24.4% not being present in the CON group; and 22.7% increasing, 76.2% decreasing and 1.2% not being present in the SARA group.

When comparing the directional dynamics between SARA_PLA and SARA_LPS groups, 40.9% of the genera behaved the same after intramammary treatment, with 67.6% increasing and 32.4% decreasing in both groups. When comparing the dynamics between CON_LPS and SARA_LPS groups, 17.7% of the genera behaved the same way after LPS injection, with 48.9% increasing and 51.1% decreasing for both groups. In contrast, 82.3% behaved differently after LPS injection, with 4.3% increasing, 76.6% decreasing, and 19.1% not being present in CON_LPS cows, and 81.3% increasing, 12.9% decreasing, and 5.7% not being present in SARA_LPS cows.

Among the single genera, four increased with a tendency (*Aerococcus*, *Trueperella*, *Kocuria*, unsas. *Rhizobiaceae)* and none decreased significantly or tendentially in the CON group during the feeding trial. During the SARA feeding, one genus increased with tendency (*Carnobacterium*), three genera decreased significantly (*Pseudomonas*, unas. *Clostridia*, *Oligella*), and 12 decreased with a tendency (*Thauera*, unas. *Saccharimonadaceae*_TM7a, *Tissierella*, Candidatus_*Soleaferrea*, *Myceligenerans*, *Phascolarctobacterium*, *Akkermansia*, *Atopostipes*, *Ruania*, *Aerococcaceae*, *Ralstonia*, *Burkholderia*-*Caballeronia*-*Paraburkholderia*) (Fig. 3, Figure S3, Additional file 1).

**Fig. 4:**
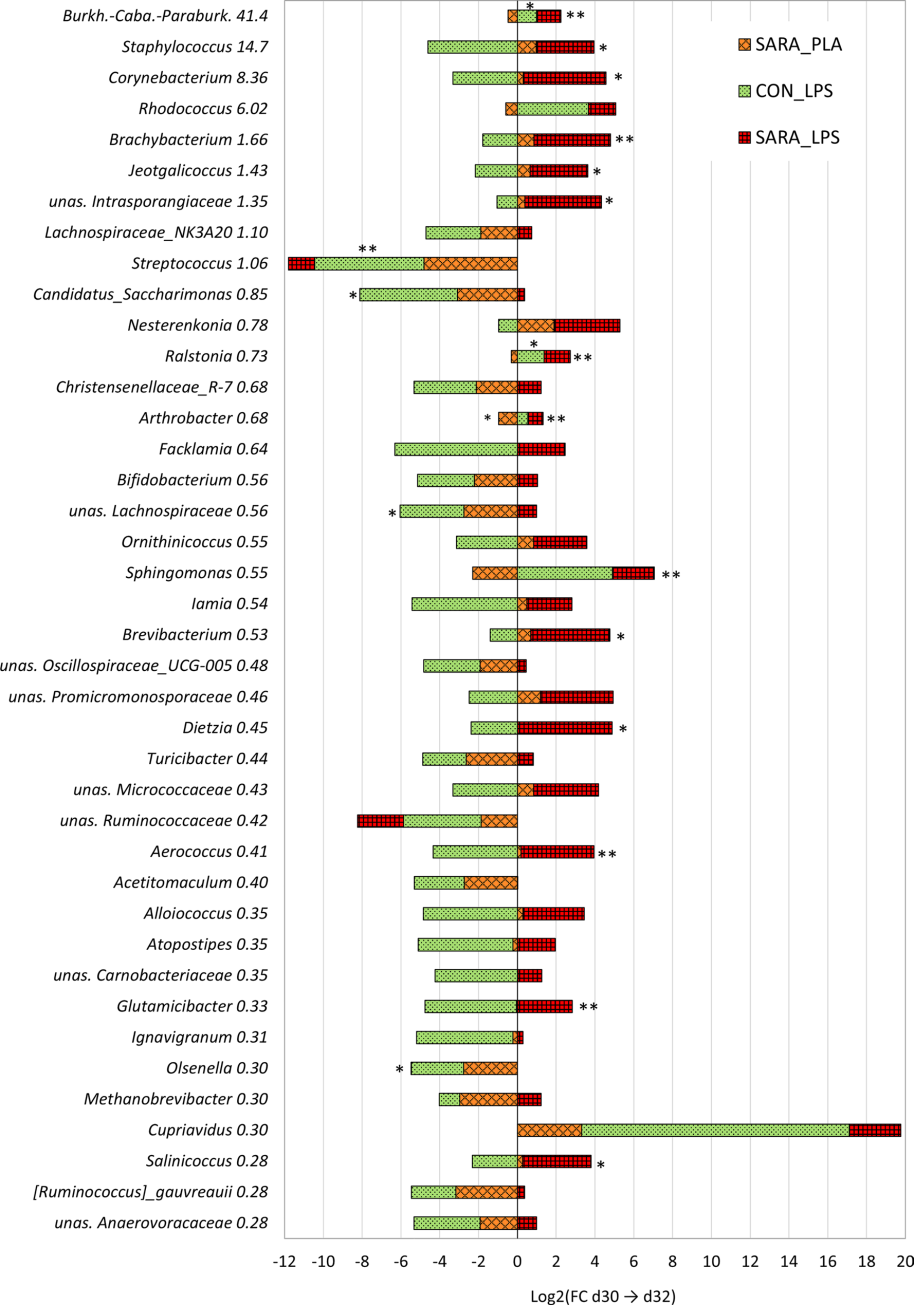
Dynamics of the 40 most abundant genera during the LPS challenge in comparison to the placebo group. The bars display the log2 of the fold change (FC) of the absolute abundance from before LPS injection (d30) to 45 h after LPS or PLA injection (d32). CON cows were on a 40% grain diet, SARA cows on a 60% grain diet. The genera are sorted from high to low overall frequency with the % reported next to the taxonomic name. unas. = unassigned; Burkh.-Caba.-Paraburk. = *Burkholderia-Caballeronia-Paraburkholderia*; unas. *Anaerovoracaceae* = *Anaerovoracaceae*_Family_XIII_AD3011; **P ≤ 0.05; *P ≤ 0.1

In the SARA_PLA group, only one low abundant genus showed a tendential increase (*Saccharopolyspora*), while only one low abundant genus (*Arthrobacter*) showed a tendential decreased after the PLA injection (Figure S4, Additional file 1). In the CON_LPS group, two genera increased tendentially after LPS injection (*Burkholderia*-*Caballeronia*-*Paraburkholderia*, *Ralstonia*), while two genera decreased significantly (*Rhizobiaceae*, *Streptococcus*), and ten decreased tendentially (*Roseburia*, *Coprococcus*, *Trueperella*, *Eubacterium coprostanoligenes*, Candidatus_*Saccharimonas*, *Syntrophococcus*, *Eubacterium brachy*, unas. *Lachnospiraceae*, *Clostridia*_UCG-014, *Olsenella*) (Fig. 4; Figure S4, Additional file 1). In the SARA_LPS group, seven genera increased significantly (*Arthrobacter*, *Burkholderia*-*Caballeronia*-*Paraburkholderia*, *Ralstonia*, *Sphingomonas*, *Glutamicibacter*, *Aerococcus*, *Brachybacterium*) and 11 by trend (*Methylobacterium*-*Methylorubrum*, *Jeotgalicoccus*, *Planococcus*, *Staphylococcus*, *Salinicoccus*, *Corticicoccus*, unas. *Intrasporangiaceae*, *Brevibacterium*, *Corynebacterium*, *Aliicoccus*, *Dietzia*) (Fig. 4; Figure S4, Additional file 1). No genus decreased significantly nor tendential in the SARA_LPS group. (Fig. 4; Figure S4, Additional file 1)

We further examined the general Gram-characteristics of the found genera in the quarter milk-samples, which revealed that 67.0% were classified as GPB, 31.5% as GNB, and 1.5% as variable or others (Archaea). To determine if the Gram-characteristics of the genera could play a role in the effect of the diet or the LPS challenge on the abundance, we compared the FC of GPB and GNB between the feeding and LPS groups using pairwise Chi^2^ test. In total, the datasets contained 2.1–3.1 times more GPB than GNB and the general increasing and decreasing trends with the feeding groups and LPS groups was similar for GPB and GNB. So did both GPB and GNB have more genera increasing with CON (74.0%; 58.6%), and more genera decreasing with SARA diet (63.2; 68.2%, respectively) (*P* < 0.001), and both GPB and GNB had more genera decreasing with SARA_PLA (64.2%; 58.6%) and CON_LPS (91.8%; 68.6%) (*P* < 0.001) and more genera increasing with SARA_LPS (83.2%; 70.4%) (*P* < 0.001). Within one feeding or LPS group, GPB and GNB showed only difference for CON_LPS, where more GPB increased than GNB (*P* < 0.001), but no intra-group difference for CON, SARA, SARA_PLA, or SARA_LPS (*P* > 0.1).

### Correlation analyses of milk components with microbial abundance and diversity

**Fig. 5:**
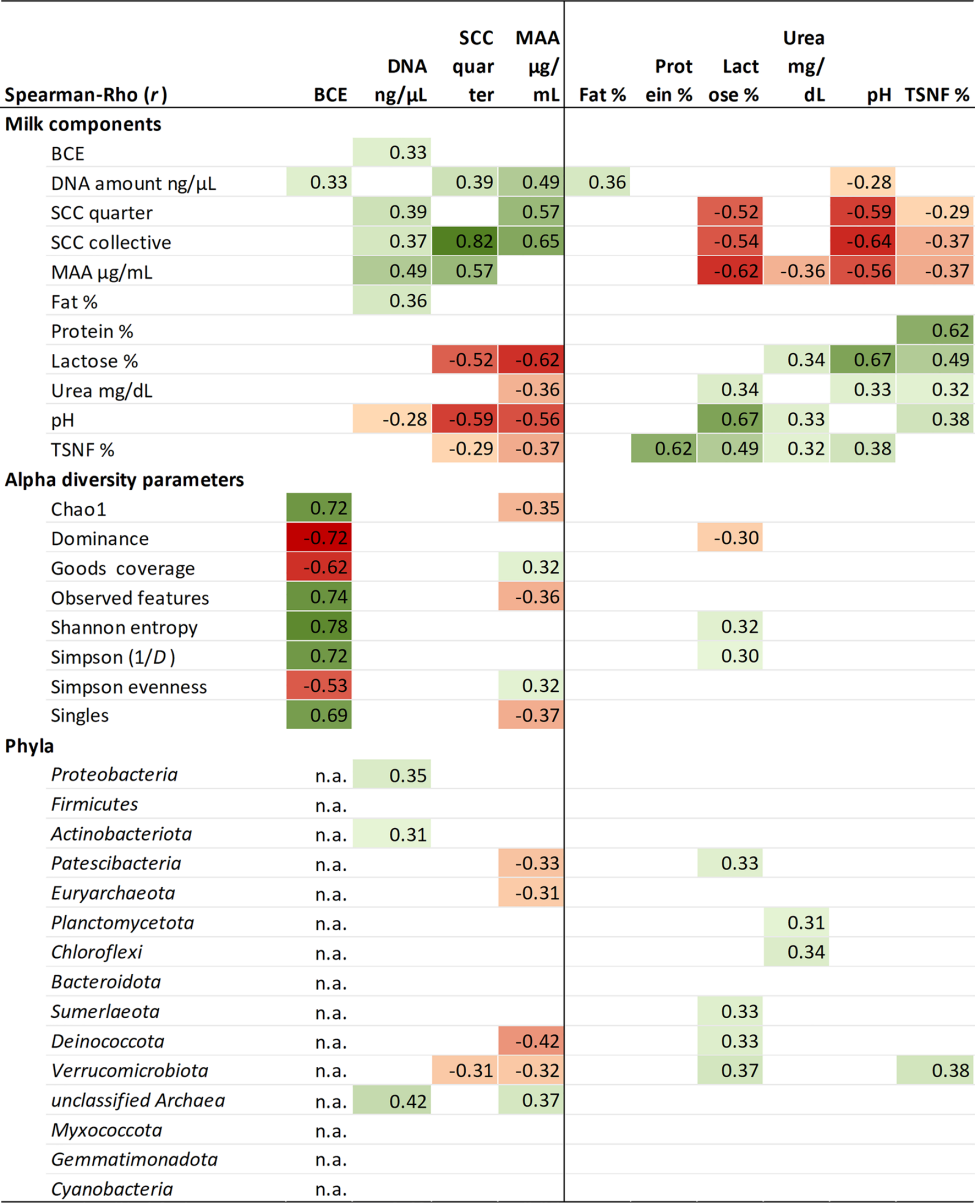
Correlation of the milk components within each other and with the alpha-diversity parameters and phyla. Only significant (*P* ≤ 0.05) and relevant (*r* |≥0.3|) correlations are shown. All samples of the experiment were included in the analysis (d-2, d30, and d32). Phyla are sorted by their frequency from high to low. Total DNA was measured using Qubit; SCC = somatic cell count; MAA = milk amyloid A; TSNF = total solids-non-fat; n.a. = not analysed, because the bacterial cell equivalent (BCE) was used to calculate absolute abundance of the phyla

**Fig. 6:**
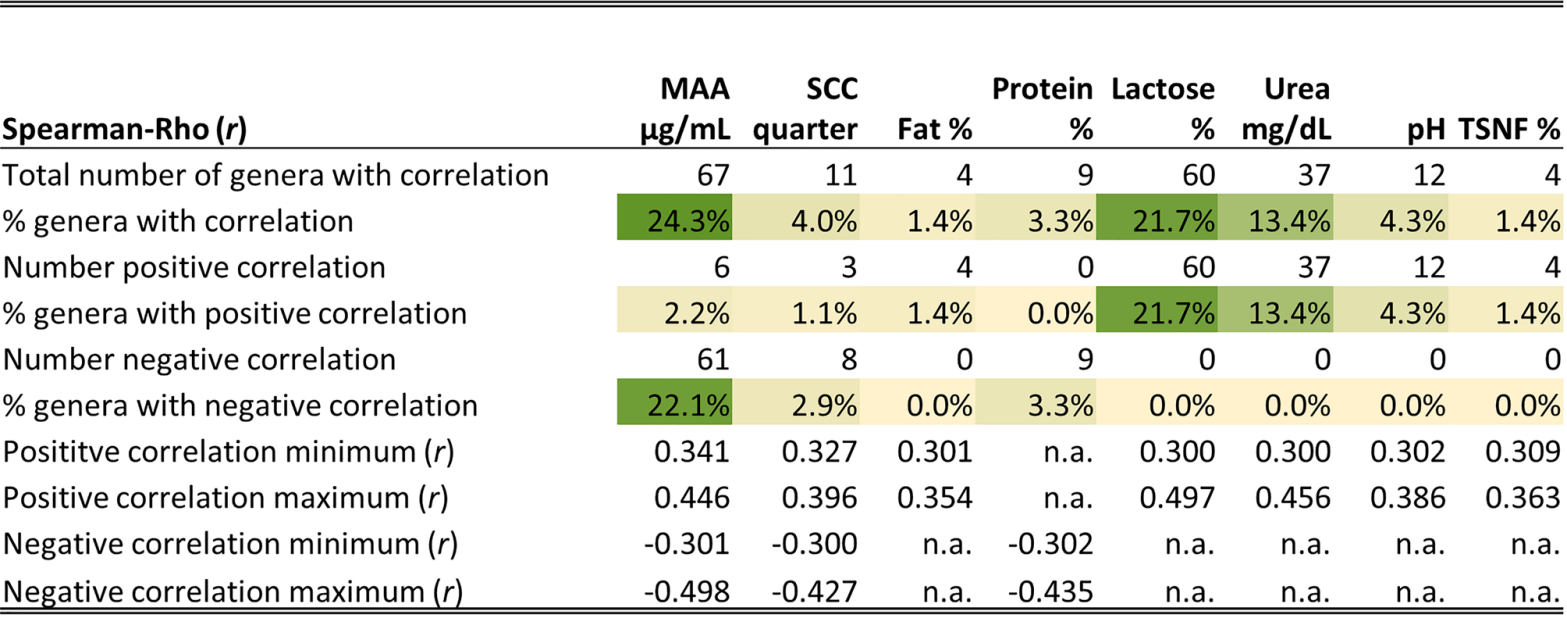
Overview of the correlation of the genera with the milk components. Summary of the significant (*P* ≤ 0.05) and relevant (*r* ≥ 0.3) correlation of all assigned genera (276) with the milk parameters during the experimental trial. The full list of correlating taxa can be found in table S3, Additional file 1. MAA = milk amyloid A; SCC = somatic cell count; TSNF = total solids-not-fat

Spearman correlation analysis was conducted among the milk components, as well as with alpha-diversity parameters, phyla (Fig. 5), and genera (Fig. 6; Table S3, Additional file 1).

Cell- and immune-related parameters showed positive correlations with each other, as did DNA amount and MAA, both of which increased with rising SCC. In contrast, milk components such as lactose, TSNF, and pH exhibited negative correlations with cell- and immune-related parameters. The alpha diversity parameters were primarily correlated with BCE, where increasing diversity and richness were associated with higher dominance but lower evenness, as BCE increased. The phyla exhibited only minor correlations with the milk components, most with MAA and lactose (Fig. 5). Of the genera, 22.1% showed a negative correlation with MAA, 21.7% showed a positive correlation with lactose, and 13.4% showed a positive correlation with urea (Fig. 6).

### Comparison between two healthy udder quarters of the same cow

**Fig. 7:**
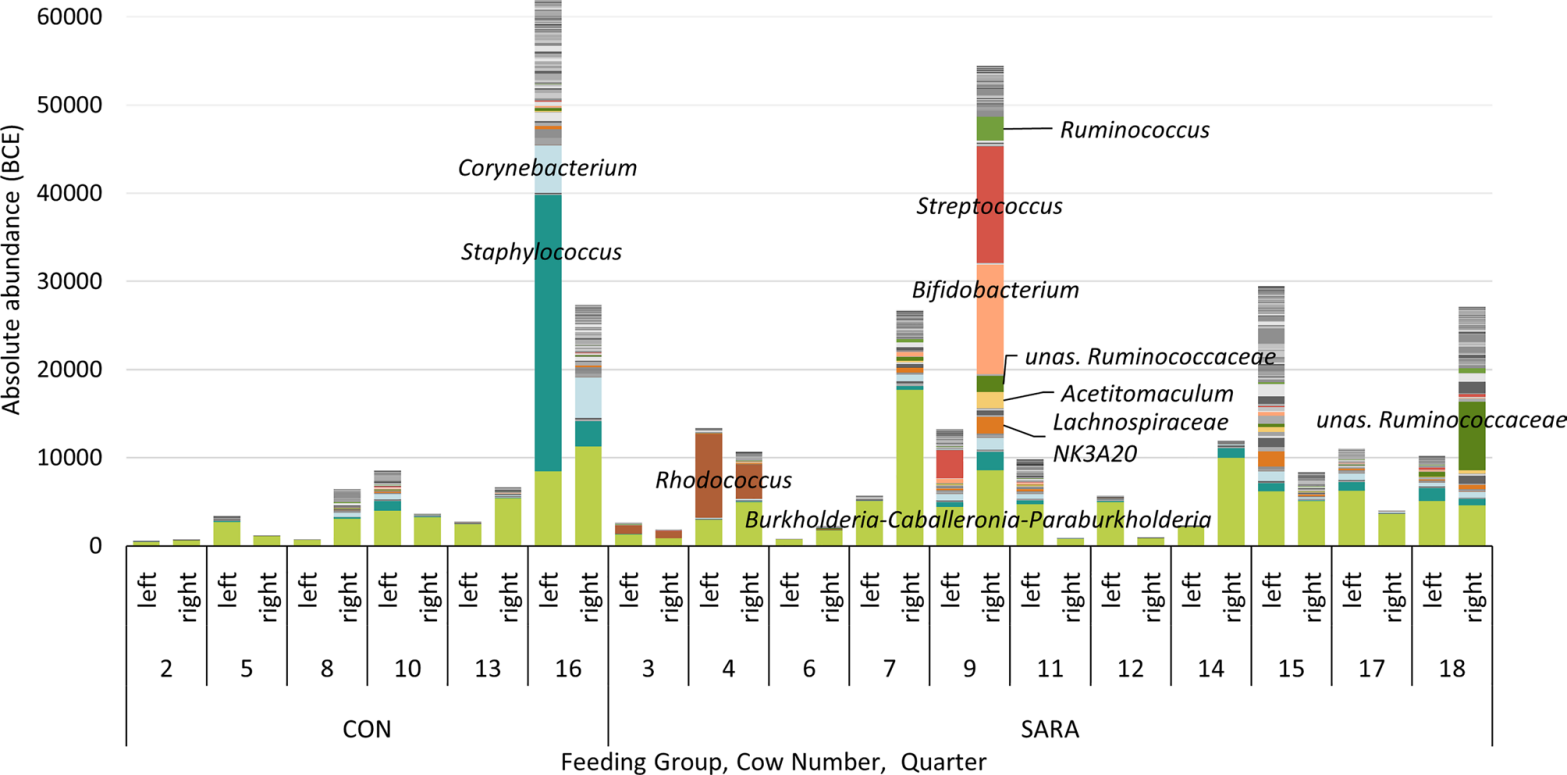
Comparison of the two udder quarters of individual cows. Absolute abundance of the bacterial communities of the left and right quarter of the 17 cows at d30. Cows were either in the moderate-grain group (CON, 40% grain) or the high-grain group (SARA, 60% grain). High-abundance genera are labelled. unas. = unassigned

Overall, the comparison of the left and right quarters for all cows did not reveal any differences for alpha-diversity parameters, the phyla, or beta-diversity. The visual inspection of the PCoA plots of Bray-Curtis dissimilarity and Weighted UniFrac distance matrices showed that the quarters of the individual cows have a similar distance to each other, but not all do pair closely (Figure S5, Additional file 1). The genera for each individual cow and her two quarters are shown in Fig. 7. For most of the cows, the two quarters have a similar absolute abundance and community distribution. Interestingly, if there is a difference in the total abundance of bacteria, there are a few, well-described genera that become dominant. These samples also inherited a higher number of different lower abundant genera than those that were generally low in BCE. There was no association between the community profile and the SCC of the individual quarters.

## Discussion

### Udder milk microbial community

Mastitis is one of the most detrimental diseases in dairy cows concerning animal welfare, production, dairy economy, and milk quality and safety [53]. While culture-dependent methods are used as diagnostic tools to identify specific known mastitis pathogens, molecular methods have revealed the presence of a diverse community even in the milk of healthy dairy cows [2]. This community changes in cows with different stages of intramammary infection [54].

In most of the studies, udder quarters defined as healthy - using clinical signs, SCC, or bacterial culture - typically have a higher diversity than those showing signs of infection or inflammation [55–57]. To get a better understanding of the individual quarter microbiome, we compared the microbial community of two quarters of the individual cows as part of our study. For most quarter-pairs, there was little variation as they were dominated by the highest abundant genus (*Burkholderia-Caballeronia-Paraburkholderia*). In some quarters, the abundance of multiple genera clearly increased, as for example *Streptococcus*, *Staphylococcus*, *Bifidobacterium*, *Ruminocccus*, or *Corynebacterium* became dominant in those samples. This aligns with our finding that, with increasing BCE, the alpha-diversity (i.e. Shannon and reciprocal Simpson) increases, while the evenness, or equal distribution of abundances, decreases. The simultaneous decrease of dominance highlights that the increase in BCE is not driven by some certain species, but an increase in the total microbial consortia.

Other than expected, we did not find any correlations of the SCC with BCE, the alpha-diversity parameters, or the abundance of phyla, and only with a few of the genera. Additionally, for the two quarters, there was no relationship between community shape and SCC status at the time of sampling. Only the PERMANOVA showed a tendency for SCC groups for Bray-Curtis and significance for Weighted UniFrac, however when visually observing the PCoA plots, the samples below or above 200,000 cells/mL overlap substantially. Further the SCC changes in this study are mainly driven by the LPS injection, which is an influencing factor on its own. Other studies have found higher diversity and lower evenness in low SCC cows [15], or a higher Shannon-index in healthy quarters compared to those with mastitis [54, 56]. However, other studies found only no or weak correlation between SCC and taxa or diversity [58, 59], or did not perform correlation analysis at all [15, 56]. Aligning, not all studies did find that animals with mastitis show microbial profiles distinct to healthy individuals [60, 61]. It seems that the immune cells measured by the SCC are not the ultimate predictor of the milk microbiome, as this frequently used parameter does not account for all infections or stages of mastitis [62]. Conversely, several genera correlated negatively with MAA, which itself exhibited a moderate positive correlation with SCC. One plausible explanation for the absence of significant correlations between SCC and the milk microbiota in our study could be the high inter-individual variability of SCC, particularly in the SARA group before LPS injection, and the LPS injection being the main influencer on SCC in our study, mimicking direct influences of SCC. Moreover, culture-based studies have repeatedly demonstrated culture-positive but low SCC quarters. Especially minor mastitis pathogens such as *Streptococcus uberis* or non-aureus *Staphylococci*, but even *Staphylococcus aureus* have been isolated from non-mastitic samples ( < 50,000 cells/mL) [63]. The diagnostic sensitivity of the conventional threshold of 200,000 cells/mL for mastitis at quarter level, remains limited for coagulase-negative *Staphylococci* (59%), minor Gram-negative bacteria (77%), and even for *Staphylococcus aureus* (85%) [1]. As highlighted in recent literature, the use of differential somatic cell count (DSCC) should be considered as a more reliable and specific indicator of inflammatory processes in future investigations [64, 65].

Summarizing this, the quarters of the same cow exhibit a similar microbial community. However, certain potential pathogens may already become dominant even in healthy, low SCC quarters, posing a risk for subclinical mastitis if the cow’s metabolism is challenged by an additional stressor [66]. In the future, this information could be used as a predictive tool for mastitis.

Another aspect to mention is that we have first described that the milk microbial community of healthy quarters is dominated by GPB. Taking culture-based studies on raw milk and mastitis pathogens, it also seems that GPB are predominant [67, 68], but culture biases need to be considered. In general, sequencing-based studies do not examine the distribution of Gram characteristics in their samples. Therefore, our study provides the first holistic overview of this feature in bovine milk samples and suggest to consider this characteristic in future studies.

### Effect of diet on milk microbial community

In order to elucidate the effect of diet, specifically moderate- and high-grain diets, on milk microbes, we compared the development of the milk community and the milk contents during a 39-days feeding trial. While beta-diversity was significantly different for CON and SARA in the PERMANOVA, alpha diversity did not show differences during the experimental time. This was also observed in other studies investigating the effect of high-grain diets on the milk bacterial community [20, 21]. However, the abundances of the members of the microbial community showed different patterns between the two feeding groups. While in the CON group 70.1% of the genera increased their absolute abundance, in the SARA group 65.3% of the genera decreased their abundance during the feeding trial. The absence of distinct clustering of experimental groups in the PCoA, could be caused by coordinated but taxon-specific and gradual shifts that are masked in multivariate ordinations. Moreover, the high inter-individual variation among cows was considered in the analysis of the single taxa with paired samples, but not in the PERMANOVA, or visual PCoA plotting.

This longitudinal effect of diet on the milk microbiome has not been described before. Zhang et al. (2015) have also reported higher relative abundances of milk microbes in a low- (40%) concentrate feeding group, compared to a high- (70%) concentrate feeding group, when applying a 2 × 2 crossover design [21]. Taxonomic and abundance comparisons with the study of Zhang et al. (2015) should be taken with caution, since it was still a Roche 454 FLX pyrosequencing approach and taxonomic assignments have changed during the last decade. They also compared their two groups only at one timepoint, and not the development of the taxa during a timespan. Nevertheless, they also found only one phylum being higher in the high-concentrate group (*Proteobacteria*), and all other 12 phyla (e.g. *Firmicutes* or *Chloroflexi*) being higher in the low-concentrate group [21], which reflects the community directions in our study. The more recent study by Hu et al. (2022), compared the milk microbiome between 40% concentrate and 60% concentrate diet groups (*n* = 8), with significantly lower alpha diversity matrices and a lower abundance in most of the phyla and genera in the milk of SARA cows at one sampling time point [22]. Mu et al. (2023), also compared the communities between 40 and 60% concentrate diet groups (*n* = 4), but only at one timepoint. They only found one genus (*Labrys*) to be higher in the 40% concentrate group, and no further significant nor biological trends on phylum and genus level [20]. Considering potential influence of host factors, such as behavior, udder and teat anatomy, immune status, or genetics on the milk microbiome [2, 69], which might even outweigh dietary effects, comparisons between groups of different animals should be performed with a high number of individuals, to milder individual host-influence. With a moderate number of cows in the SARA (*n* = 12) and CON (*n* = 6) group, our study describes for the first time a longitudinal effect of the diet on the milk microbiome in the same animal, using paired sample analysis. This allows to correct for host-specific influences. Therefore, caution should be taken when comparing different studies with different study designs.

To determine whether individual taxa respond differently to the two diets, we compared the behavior of single genera in the two dietary groups. The majority (63.0%) behaved differently under CON versus SARA feeding during the experimental period, suggesting that the underlying mode of action is driven by the diets for most of the genera, rather than a simple time effect [58].

As mentioned before, there could now be various explanations for these dynamics related to feeding. During SARA, it is well known that bacterial diversity and abundance decrease in the rumen [70], and our accompanying research found a decrease in both ruminal and fecal pH and the microbial diversity in the feces of these cows [27, 28]. Assuming a constant interaction between the mammary gland and the environment, changes in the microbes shed by the cows may influence the milk microbiome. A reduced microbial abundance in the milk during SARA feeding corresponds with a similar reduction in the rumen and the feces under SARA conditions [28, 70]. Historical data on mastitis clearly indicate that the environment plays a major role in infection events [53]. Network analysis has shown that feces may contribute microbes to the milk [71]. However, dominant bacterial taxa remain distinct across the rumen, feces, and milk [15]. This aligns with our findings, when comparing the milk microbiome with the fecal microbial community during the experimental trial [28]. Between the milk and the feces samples, only 5 of the 14 phyla are shared. While the milk is dominated by *Proteobacteria* (47.3%), *Firmicutes* (28.1%), and *Actinobacteriota* (22.3%), the feces were dominated mainly by *Firmicutes* (82.45%), followed by *Proteobacteria* (11.41%) and *Bacteroidota* (3.0%). The dynamics of the phyla are different during the feeding trial, so do for example *Proteobacteria* increase in the feces but decrease in milk during SARA [28]. Among the 100 most abundant fecal OTUs, 63% showed a decrease in relative abundance in the SARA group and 58% showed an increase in the CON group during the feeding trial [28]. Although similar overall dynamics during the feeding trial, the milk samples shared only ten genera (unassigned *Ruminococcaceae*, *Ralstonia*, *Mathanobrevibacter*, *Ruminococcus*, *Clostridium*, *Sphingomonas*, *Methanosphaera*, *Akkermansia*, *Succiniclasticum*, *Ruminobacter*, *Succinivibrio*) with the highly abundant fecal genera and those do have different abundances and behaviors during the feeding trial [28]. Therefore, feces as part of the cow-environment, seem to have a rather minor effect in our study, as also shown in other studies [15, 54]. Feces as representation of the gut microbiota, share common taxa and exhibit similar dynamics; however, they cannot solely explain the gut-mammary gland axis theory. Different body sites in cows are known to harbor shared but also individual bacterial communities [69].

An endogenous translocation of bacteria, or at least parts of them, via the gut-mammary gland axis is currently a highly discussed topic [72]. In cattle health, the translocation of opportunistic pathogens during high-grain feeding has long been associated with liver abscesses [73]. There are ongoing efforts using mouse models to prove the concept that the gut microbiome influences mammary gland health [13]. While the current dataset does not explain any causal relationships, it clearly suggests that, besides the exogenous route, bacteria could be translocated to the mammary gland because of changes in abundance due to high-grain feeding. Since we used a Matrix-lysis protocol to extract DNA from the milk-samples, we can argue that the genera found are from intact bacterial cells, meaning they have been able to enter the mammary gland as at least viable, if not active microorganism, as free DNA would be removed with this protocol [31]. We also investigated Gram-characteristics, which showed that directional changes within the CON and SARA groups were similar for GPB and GNB. In the healthy rumen it is assumed to harbors more GPB [70], which could translocate to the mammary gland.

The SARA cows showed an increase in lipopolysaccharide binding protein, serum amyloid A, and haptoglobin in the blood by day 8 of the experiment [17]. The cows seemed to have adapted systemically to the high-grain feeding by day 30, because blood values of acute phase response decreased by that timepoint again. However, there remains the possibility that rumen-derived LPS translocation still influenced the local immune response and mammary epithelial integrity on day 30, thereby altering the milk microbiota [2, 74, 75]. Unfortunately, we did not measure other immune markers, or for example histamine in this study. Previous work has shown that histamine increases in the rumen with an intermitting 60% grain feeding model [76] and that translocated histamine activates inflammatory and protein synthesis genes in the mammary gland [77]. Hu et al. (2022) have shown that SARA, induced by 60% concentrate feeding, leads to a significant increase of the blood-milk barrier permeability. This was shown by histopathological changes and decreased expression of tight-junction proteins in the mammary gland tissue, and higher levels of IgG and lactate dehydrogenase, which are blood-derived proteins that are found in higher amounts when epithelial permeability is increased [22, 78]. Further, inflammatory cytokines were higher in the milk of SARA cows than in control cows [22]. The local inflammatory response triggered by systemic SARA effects is therefore likely one of the driving factors in shaping the milk microbiome also in our study – either by the immunogenic activity or translocating bacteria via the impaired epithelium.

Another aspect to consider is that during SARA the macro- and micronutrient composition of the milk may have changed, potentially shaping the microbiome [77, 79]. We found that a large portion of the genera, none of which included known mastitis pathogens, correlated positively with lactose. Although the changes in lactose were small but statistically significant, reduced lactose content during SARA might have impaired the growth of commensal bacterial groups, as many species can utilize lactose as carbon source [80]. The dominance of known mastitis pathogens could have also caused competition for lactose and a decrease in commensal bacteria. This finding is underlined by culture-based studies, where subclinical mastitis pathogens were higher in samples with lower lactose content in the milk [81]. The role of lactose utilization by commensals and pathogens in the bovine udder should be further investigated to better evaluate lactose as a potential biomarker for subclinical mastitis.

### Effect of lipopolysaccharide injection on milk microbial community

When LPS was injected, there was a clear increase of SCC and MAA in the milk in both the CON_LPS and SARA_LPS cows, accompanied by a strong systemic immunogenic reaction, such as significantly increased serum acute-phase proteins and LPS-binding protein [17], which suggest a disruption of the blood-milk barrier and the appearance of LPS in the systemic circulation. Mouse models have shown that the intramammary application of LPS disrupts the blood-milk barrier by altering claudins in the epithelial tight junctions [82, 83], increasing the paracellular transfer of blood and milk components. Consequently, serum antibodies appear in higher amounts in the milk following LPS injection. Usually IgG_1_ is actively transported, whereas IgG_2_ leaks passively into the milk during inflammation [84]. Given its bacterial binding capacity, IgG_2_ might also interact with commensal bacterial during the onset of LPS-induced inflammation, potentially influencing the composition of the milk microbiome.

Interestingly, a large part (22.1%) of the genera showed a negative correlation with MAA, while only 2.2% exhibited a positive one. MAA, as an acute-phase protein, is a part of the innate-immune system and is produced by the mammary-gland epithelium as one of the first defense mechanisms against microorganisms [85]. There is evidence that MAA attracts B-Lymphoblasts, which are as plasma cells responsible for antibody production [86]. The correlation analysis underlines that the production of MAA, along with other immune components triggered by the LPS injection [87], resulted in the reduction of microbes in the udder [88]. While in the placebo group the trend of the SARA diet continued, the dynamics of the microbes changed in the LPS groups. The increasing trend observed during the CON feeding was reversed and we assume that the high level of MAA after LPS injection led to a decrease in abundance in the CON group [85]. This effect was particularly pronounced for GPB, where 91.8% of the genera decreased. This finding is somehow contradicting to what we know about the immune cascade following pathogen infection, as the trigger was LPS, a solely GNB-antigen. For example, TNFα has been shown in vitro to react only to LPS and not to antigens from GPB [26]

In comparison to the dietary effect, the SARA_LPS group now showed increasing abundances for most of the taxa. To the authors’ best knowledge, there is currently no literature investigating the direct effect of LPS molecules on bacteria, which could partly explain these findings. Furthermore, only one study so far has examined milk microbial shifts after LPS injection in healthy cows, but it reported a disproportionately high level of contamination in their samples, rendering its findings difficult to interpret [89].

It is more likely that the systemic effects of SARA and the local impact of LPS interacted with each other. Changes of small molecules, histological alterations, or the immune system response may have contributed to this interaction [90]. For instance, the SARA_LPS cows showed higher overall MAA values than the CON_LPS cows. However, the reaction for MAA from before to after LPS injection was stronger in the CON_LPS cows than in the SARA_LPS cows. Accordingly, the SARA_LPS group had mostly similar levels in their blood metabolome like the SARA_PLA group, and CON_LPS differed significantly from those two groups [17]. Some studies have already shown that pre-exposure to LPS can milder the immunogenic effects of subsequent *E. coli* infections [91]. The pre-exposure of our SARA cows due to endogenous LPS from the gastrointestinal tract might have already activated the local udder-immune system, which is supported by the numerical increase of MAA, the acute phase proteins in the blood during SARA, and the metabolome shifts [17]. SARA also leads to a disruption of the epithelial barrier in the mammary gland [22], with the discussed consequences of compound translocation. Further, metabolic response was already given in SARA cows before the LPS injection, shown by lower Ca, NEFA, BHBA, and higher AST in the blood [27]. In contrast, the CON cows were completely naive to a metabolic and immunogenic challenge. The strong immune response in the CON cows after LPS injection could have caused the reversed effect of abundance, while the microbial community in SARA_LPS cows was already accustomed to immunogenic pressure and was better established when they faced a second challenge with LPS [91]. The lactose content in the SARA_LPS group increased slightly more than in the SARA_PLA and CON_LPS groups, which could have provided sufficient nutrients for the bacteria to increase their abundance in this group [80] and explains the large amount of correlation between bacterial abundance and lactose content. In future studies, integrity of the blood-milk barrier and immunogenic reactions in the udder should be assessed in more detail, for example by measuring serum albumin, cytokines, immunoglobulins (IgGs), or lactate dehydrogenase in the milk [78, 92].

This study is the first to investigate the specific influence of LPS on the milk microbial community in dairy cows during a SARA feeding model. The triggered local immune and micronutrient responses decreased bacterial abundance in the CON_LPS cows. However, the pre-exposure to endogenous LPS in SARA cows may have prepared the microbial community to increase in abundance when LPS was intramammarily applied in the SARA_LPS group.

### Limitations of the study

The use of DNA-based amplicon sequencing of the 16S rRNA gene does not allow any conclusions about the viability or activity of the identified bacterial groups. We acknowledge that numerous factors may influence the results, including the sampling process, sample transportation, freezing, sample preparation, DNA extraction, library preparation, sequencing, and the bioinformatic pipeline, despite of many benchmarking efforts [93, 94]. Similarly, the resolution of the 16S rRNA gene sequencing is limited - further constrained by the fact that here only two variable regions were sequenced - and may not distinguish closely related organisms. In future studies, full length 16S gene sequencing or metagenomic approaches would provide higher taxonomic resolution. Taxonomic assignments depend on available databases (e.g., SILVA), which, despite rigorous and ongoing maintenance, may be still incomplete for certain underexplored taxa and environments. These restraints might have also influenced the proportion of GNB and GPB in our dataset, a characteristic that should be investigated in more detail in other studies.

These limitations of taxonomic gene amplicon sequencing, among others, necessitate cautious interpretation of the results, particularly concerning the role of the individual bacterial groups. Comparisons with other studies are also challenging due to differences in workflows and metrics (e.g. classic 95% OTU vs. ASV). Additionally, the small sample size in this study is a known limitation. A larger sample size would strengthen the findings, but constraints inherent to experiments involving large animals must be acknowledged. To minimize animal use, a CON_PLA group was not included, as no effects were expected in such a group. In future studies, direct or indirect assessment of microbial viability, by targeted culturing methods or dead-life staining, microbial function, via metatranscriptomics or metabolite profiling, microbial-host interaction, via mammary epithelial gene expression or bacterial translocation assays, would help to create deeper functional understanding. As tissue biopsies and the use of labelled bacteria are invasive and not feasible in lactating cows producing milk for human consumption, this was not performed in this study. Non-invasive alternatives, such as RNA extraction from milk fat globules, fatty acid profiles, or lipolytic activity could be explored in future studies. Despite these limitations, our results reveal meaningful patterns in microbial composition and correlations with mammary immune markers, providing a foundation for future work on bacterial viability, function, host–microbe interactions, and microbial migration to the mammary gland.

## Conclusions

In this work, we demonstrated that, based on a SARA feeding model, the microbial abundance in the milk of dairy cows increased when cows were fed a moderate- grain diet (40%) and decreased when challenged with a high-grain diet (60%). This suggests that dietary composition plays a crucial role in shaping milk microbial community, potentially through changes in rumen fermentation, immune responses, impaired blood-milk barrier, or microbial translocation.

When LPS, a potent endotoxin, was injected into the quarters, the effect of diet was reversed. In the CON_LPS cows, bacterial abundances decreased, while in the SARA_LPS cows, the abundances increased. The reversing effect might have occurred because the local immune response in the udder was already triggered due to SARA effects, allowing the microbial community to adapt to the immunogenic response and increase in abundance. In contrast, the CON_LPS group, with both the immune system and microbes being naïve to any challenge, caused a strong reversing reaction.

The SARA-Placebo group continued the trend of decreasing abundances, as observed prior to injection. We also compared two healthy quarters of the same individuals, which mostly showed similar bacterial communities. However, when there was an increase in BCE it typically involved one dominant species alongside many low abundance ones, without a change in SCC. This was supported by the lack of correlation between microbial genera or alpha diversity and SCC. Although the PERMANOVA showed significance for a SCC-threshold of 200,000 cells/mL, the visual observation of the PCoA ordination did not reveal a clear separation of the samples. This raises questions about the role of SCC in reflecting the microbial community in the udder. Nevertheless, the strong negative correlation with MAA lets us conclude that the immune system plays a major role in shaping the microbial community in healthy quarters, particularly during different diets and endotoxin challenge.

## Electronic supplementary material

Below is the link to the electronic supplementary material.

Additional File 1

Additional File 2

## Data Availability

Sequence data that support the findings of this study have been deposited in the European Nucleotide Archive with the primary accession code PRJEB87653. The bioinformatic pipeline used in this article is available in the github repository of N.M.Q. [95].
