# Supplementary material for: Dynamics of the milk microbial community during subacute ruminal acidosis with or without intramammary lipopolysaccharide challenge in dairy cows
Source: Anim Microbiome. 2026 Feb 26;8:24. doi: 10.1186/s42523-025-00499-5 (PMC12937525; doi:10.1186/s42523-025-00499-5)

Additional File 1

Dynamics of the milk microbial community during subacute ruminal acidosis with or without intra-mammary lipopolysaccharide challenge in dairy cows

Viktoria Neubauer, Siska Aditya, Narciso M. Quijada, Stefanie Urimare Wetzels, Monika Dzieciol, Poulad Pourazad, Qendrim Zebeli, Evelyne Selberherr

Figure S1. Experimental trial. 18 Simmental cows were enrolled in the study. After a 7-day Baseline period, 12 cows were assigned to the high-grain (60% grain; SARA) feeding group, 6 cows to the control moderate-rain (40% grain, CON) feeding group. Baseline milk samples were collected on day -2 from the left front quarter. On day 30, milk samples were taken from both the left and right front quarter before the lipopolysaccharide (LPS) challenge. Six cows from each the CON and SARA group were intramammarily injected with 50 µg of *E. coli* LPS. The remaining 6 cows from the SARA group received a placebo (NaCl; PLA). Milk samples were collected on day 32 from the left front quarter, approximately 45h after the challenge.

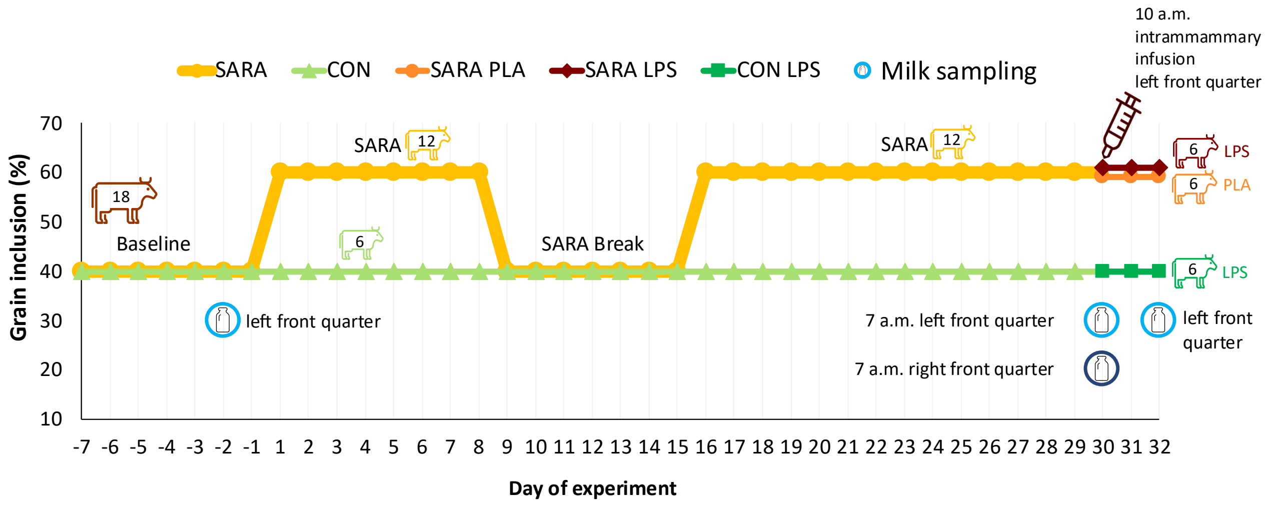

Table S1. Absolute values of the milk parameters at d-2 (Baseline, all cows CON diet, 40% grain), d30 in the CON group (n=6) and the high-grain diet group (SARA, 60% grain; n=12), which is also before intramammary treatment; on d32, 45h after lipopolysaccharide (LPS; n=12) or NaCl (Placebo, PLA n=6) injection. BCE = bacterial cell equivalent; total DNA measured by Qubit; SCC = somatic cell count; MAA = Milk-Amyloid-A; TSNF = total

**Milk parameter**	d-2				d30				d32					
	CON	SD	SARA	SD	CON	SD	SARA	SD	SARA_PLA	SD	CON_LPS	SD	SARA_LPS	SD
BCE	5655	5922.39	11344	11042.66	12929	24150.68	8923	7785.67	8385	9468.85	7569	7193.28	19337	19088.64
DNA ng/µL	0.44	0.36	0.73	1.22	0.53	0.87	1.35	3.77	0.43	0.46	1.98	1.74	5.88	5.78
SCC (×1000)	22	18.75	613	1998.10	27	27.27	116	293.49	4076	9897.27	6169	4233.72	7789	5100.22
MAA µg/mL	0.31	0.44	0.79	2.18	0.12	0.11	1.73	5.35	0.18	0.19	490.10	445.90	701.08	456.10
Fat %	2.45	0.41	2.40	0.91	1.80	0.45	1.22	0.58	1.79	0.68	2.48	0.48	2.59	0.81
Protein %	3.17	0.20	3.28	0.34	3.13	0.27	3.43	0.39	3.47	0.50	3.13	0.27	3.80	0.60
TSNF %	8.91	0.25	8.46	1.54	8.72	0.20	8.67	1.28	9.16	0.44	8.47	0.26	8.95	0.33
Urea mg/dL	23.25	7.07	19.25	5.55	16.70	7.51	22.51	5.38	23.95	6.94	15.74	6.67	20.50	7.08
Lactose %	5.04	0.18	4.89	0.56	4.88	0.20	4.59	1.07	5.00	0.10	4.65	0.42	4.45	0.40
pH	6.58	0.08	6.54	0.10	6.54	0.06	6.44	0.27	6.52	0.05	6.48	0.10	6.40	0.10

Table S2. Alpha diversity matrices displayed as fold change (FC) from day-2 (Baseline, 40% grain diet) to day 30 of the experiment showing the effect of diet (40% grain, CON; 60% grain, SARA), and the FC from before the injection (d30) to 45 hours after injection (d32) of lipopolysaccharides (LPS) or placebo (PLA; sterile NaCl) into the mammary gland.

	FC d-2 to d30				FC before to after injection					
**Alpha diversity matrix**	**CON**	***P*-value**	**SARA**	***P*-value**	**SARA_PLA**	***P*-value**	**SARA_LPS**	***P*-value**	**CON_LPS**	***P*-value**
**Chao1**	1.2	0.50	1.0	0.43	1.0	0.50	0.9	0.50	0.5	0.16
**Dominance**	1.1	0.42	0.9	0.34	1.1	0.50	0.9	0.50	1.0	0.50
**Goods coverage**	1.0	0.34	1.0	0.26	1.0	0.50	1.0	0.28	1.0	0.28
**Observed features**	1.2	0.50	1.0	0.48	1.0	0.50	0.9	0.42	0.5	0.16
**Shannon entropy**	1.0	0.34	1.0	0.52	1.0	0.50	1.1	0.42	0.9	0.28
**Simpson (1/*D*)**	1.0	0.42	1.0	0.34	1.0	0.50	1.0	0.50	1.0	0.50
**Simpson evenness**	1.4	0.42	0.8	0.43	1.1	0.22	1.4	0.11	0.7	0.22
**Singles**	1.2	0.50	1.2	0.22	1.0	0.50	0.8	0.28	0.5	0.16

Figure S2. Beta diversity matrices Bray-Curtis dissimilarity and Weighted UniFrac distance for milk samples taken a) on d-2 (Baseline) and d30 for the control group (CON, 40% grain) and high-grain diet group (SARA, 60% grain), for b) the milk samples after intramammary treatment on d32 with lipopolysaccharides (LPS) or placebo (NaCl, PLA), and for c) all milk samples taken in the experiment for the somatic cell count (SCC) measured.

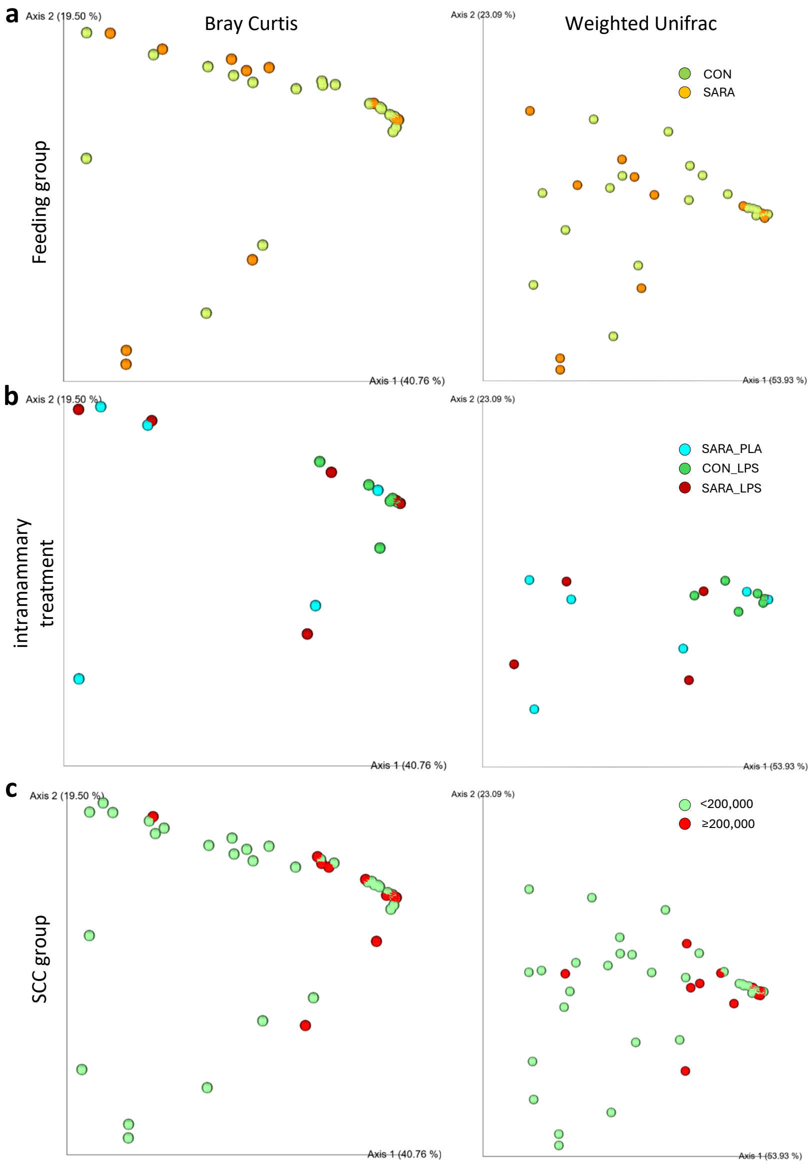

Figure S3. Dynamics of the genera with the feeding model from day -2 to day 30. Log2 fold change (FC) of the absolute abundance of all genera from the Baseline (day -2, 40% grain) to d30 in (**a**) CON cows, which received continuously a 40% grain, and (**b**) SARA cows, which were challenged with an intermittent 60% grain feeding regimen. The y-axis displays the -log10 of the P-value for changes from d-2 to d30, with the horizontal line at 1.3 for P = 0.05 and at 1.0 for P = 0.1.

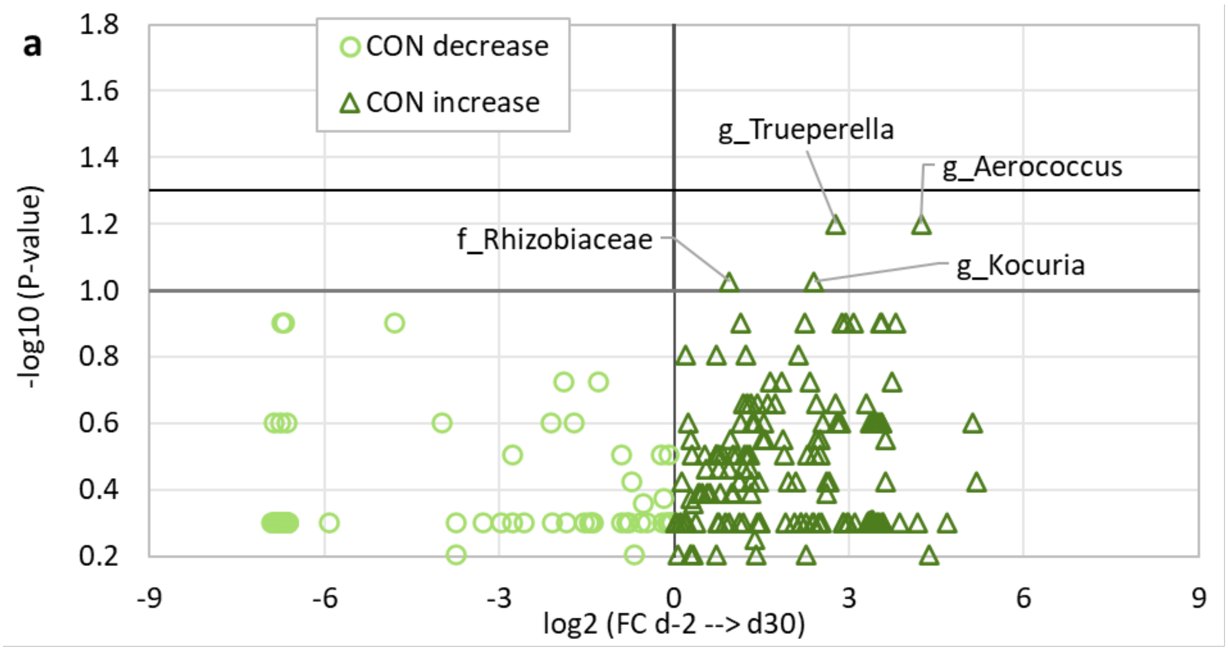

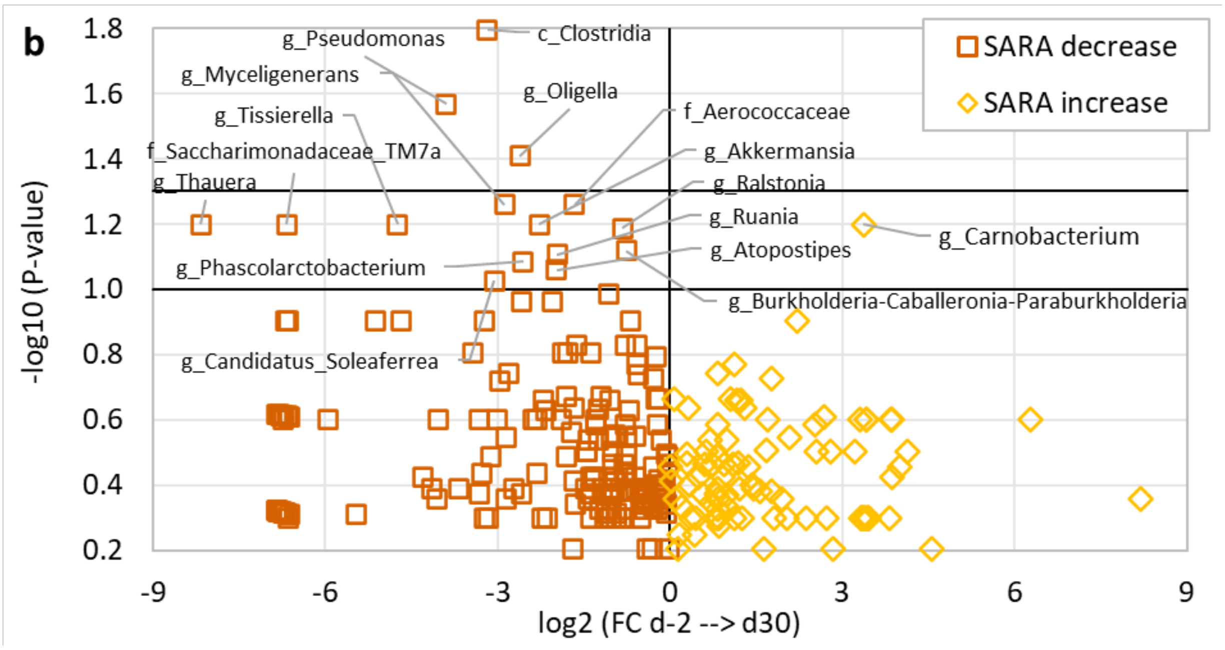

Figure S4. Dynamics of the genera during LPS challenges compared to the placebo group. Log2 fold change (FC) of the absolute abundance of all taxonomic groups from before (d30) to after (d32) LPS injection in (**a**) CON (40% grain), (**b**) SARA cows (60% grain) or (**c**) placebo (PLA) injection in SARA cows. The y-axis displays the -log10 of the *P*-value for changes from d-2 to d30, with the horizontal line at 1.3 for *P* = 0.05 and at 1.0 for *P* = 0.1.

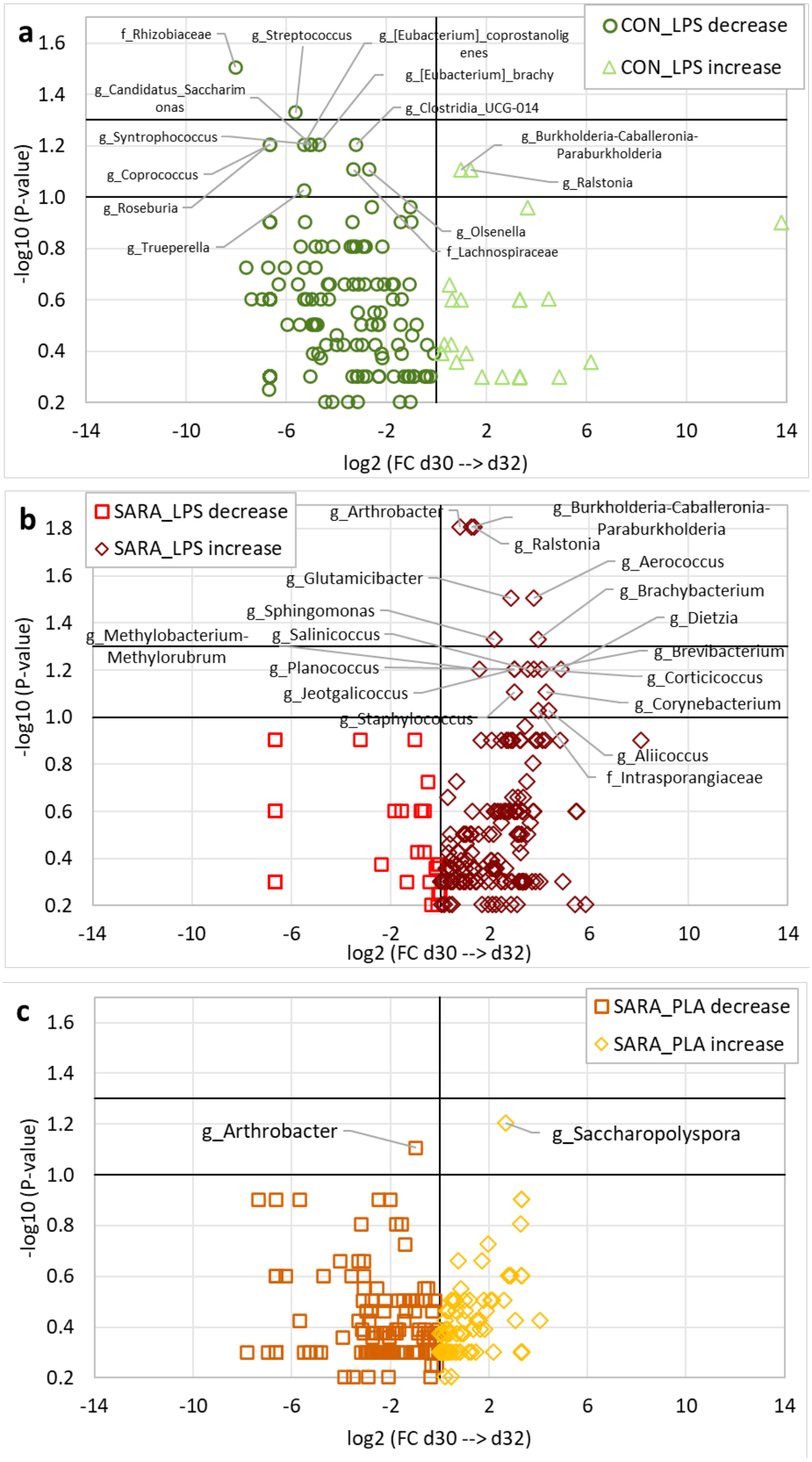

Table S3. List of the assigned genera that showed a significant ($P \leq 0.05$) and relevant ($r \geq |0.3|$) correlation with the milk parameters during the experimental trial. unas. unassigned; MAA Milk-Amyloid-A; TSNF total solids not fat.

**Spearman-Rho (*r*)**	**MAA µg/mL**	**SCC quarter**	**Fat %**	**Protein %**	**Lactose %**	**Urea mg/dL**	**pH**	**TSNF %**	**Abundance in dataset (%)**	**Number of correlations**
Streptococcus	-0.352								1.06	1
Candidatus_Saccharimonas	-0.327				0.323				0.85	2
Christensenellaceae_R-7	-0.351				0.362				0.68	2
Facklamia						0.302			0.64	1
unas.Lachnospiraceae	-0.339								0.56	1
unas.Oscillospiraceae_UCG-005	-0.344				0.366				0.48	2
unas.Micrococcaceae						0.300			0.43	1
unas.Carnobacteriaceae					0.309				0.35	1
Glutamicibacter						0.328			0.33	1
Ignavigranum					0.312				0.31	1
Methanobrevibacter	-0.310								0.30	1
Cupriavidus	0.349								0.29	2
unas.Anaerovoracaceae_XIII_AD 3011	-0.334								0.28	1
[Eubacterium]_hallii	-0.367								0.26	1
Nocardioides					0.304				0.25	1
Aeromicrobium						0.313			0.24	1
Ruminococcus	-0.321								0.22	1
Phreatobacter	0.446	0.396							0.22	3
unas.Rhizobiaceae						0.316			0.20	1
unas.Caulobacteraceae				-0.355					0.20	1
unas.Oscillospiraceae_NK4A214	-0.439				0.387	0.316			0.17	3
Clostridia_UCG-014	-0.329				0.348				0.17	2
[Eubacterium]_brachy	-0.419				0.354			0.309	0.16	3
unas.Aerococcaceae	-0.334				0.302				0.16	2
unas.Micrococcales	-0.301								0.14	1
[Eubacterium]_coprostanoligenes	-0.425	-0.301			0.404				0.13	3
unas.Anaerovoracaceae	-0.305								0.12	1
Flaviflexus					0.308				0.11	1
unas.Oscillospiraceae	-0.359				0.348				0.093	2
unas.Microbacteriaceae	-0.382				0.497	0.391			0.084	3
Lactobacillus						0.302			0.082	1
unas.Erwiniaceae				-0.313					0.081	1
[Eubacterium]_nodatum	-0.322								0.078	1
Monoglobus					0.376				0.072	1
Sumerlaea					0.330				0.069	1
Marvinbryantia	-0.329					0.322			0.068	2
Dorea	-0.421				0.387				0.061	2

unas.Thermomicrobiales_JG30-KF-CM45						0.315			0.058	1
Trueperella	-0.358								0.056	1
unas.Ilumatobacteraceae					0.306	0.323			0.055	2
unas.Eggerthellaceae	-0.382								0.053	1
Enteractinococcus					0.348				0.053	1
unas.Bifidobacteriaceae						0.339			0.052	1
Cellulosilyticum	-0.330								0.051	1
Howardella	-0.306					0.313			0.051	2
Frisingicoccus	-0.336				0.415				0.050	2
unas.Xanthomonadaceae	-0.368	-0.300			0.449	0.325	0.307		0.049	5
Saccharimonadales						0.344			0.047	1
Lachnospiraceae_FE2018	-0.356				0.308	0.320			0.046	3
Devosia					0.357	0.456			0.044	2
Gulosibacter						0.374			0.043	1
unas.Lactobacillales	-0.461				0.476				0.041	2
unas.Actinobacteria						0.366			0.039	1
Coprococcus	-0.373				0.327				0.038	3
Reyranella	0.412	0.328							0.036	3
Gallicola					0.332				0.036	1
Aeriscardovia	-0.498				0.375	0.333	0.307		0.035	4
Jeotgalibaca					0.310				0.032	1
Luteimonas					0.317				0.031	1
Phyllobacterium	0.414								0.028	1
unas.Anaerovoracaceae_XIII_UCG-001	-0.398								0.027	1
Planococcus						0.300			0.026	1
Murdochiella	-0.364								0.026	1
Timonella					0.310				0.025	1
Oligella	-0.313				0.443		0.328		0.024	3
Roseburia	-0.366	-0.304							0.023	2
d_Archaea	0.366								0.021	1
unas.Eggerthellaceae_DNF00809	-0.435								0.020	2
Shuttleworthia	-0.360				0.347	0.311			0.019	3
Jonesia						0.344			0.018	1
Truepera	-0.420				0.334				0.018	2
Sanguibacter				-0.318	0.347		0.358		0.017	3
Peptoniphilus					0.300				0.017	1
Lachnospiraceae_UCG-010						0.322			0.017	1
unas.Planococcaceae	-0.358								0.016	1
Globicatella				-0.364	0.434				0.015	2
unas.Butyricicoccaceae_UCG-009	-0.331				0.317				0.015	2
Allorhizobium-Neorhizobium-Pararhizobium-Rhizobium					0.308				0.014	1
Enterorhabdus	-0.450	-0.326			0.348				0.014	4
Gemmobacter				-0.336					0.014	1

Fastidiosipila					0.313				0.013	1
Ruania	-0.308								0.013	1
Enterococcus					0.303		0.385		0.013	2
unas.Solirubrobacterales					0.352	0.348			0.013	2
unas.Clostridia	-0.339								0.012	1
unas.Thermomicrobiales					0.417				0.012	1
unas.Nitrosomonadaceae_MND 1					0.309				0.012	1
unas.Kiritimatiellae_WCHB1-41								0.341	0.011	1
Negativibacillus	-0.311								0.011	1
Weissella					0.314	0.416			0.011	2
unas.Oscillospiraceae_UCG-002	-0.400				0.416				0.010	3
unas.Atopobiaceae	-0.327								0.010	1
Paeniglutamicibacter	-0.303								0.010	1
Lachnospiraceae_UCG-001	-0.315				0.412				0.010	2
Arenimonas					0.371	0.329			0.010	2
Georgenia	-0.417			-0.302	0.406	0.360			0.010	4
Terrisporobacter	-0.402								0.009	1
Agathobacter						0.319			0.009	1
Peptococcus					0.435				0.008	1
Fimbriiglobus	0.341	0.327							0.008	3
Anaerovibrio	-0.309								0.008	1
Methanosphaera	-0.368								0.008	2
Tissierella					0.416				0.007	1
Phascolarctobacterium	-0.343				0.369				0.007	2
Solobacterium	-0.395	-0.330			0.341	0.350			0.007	4
unas.Gammaproteobacteria	-0.302								0.007	1
Pseudomonas			0.332						0.007	1
Lachnospiraceae_UCG-002	-0.370				0.349	0.380			0.006	4
Proteiniclasticum					0.422	0.362	0.314		0.006	3
unas.Rhodobacteraceae				-0.359		0.311	0.311		0.006	3
Bacteroides									0.005	1
Erysipelatoclostridium					0.331		0.316		0.005	2
Leucobacter				-0.354	0.358				0.005	2
Eubacterium	-0.432	-0.339			0.406				0.005	4
Anaerofustis	-0.319						0.302		0.005	3
Akkermansia	-0.339	-0.312			0.417			0.318	0.004	4
Alkalibacterium			0.313						0.004	1
Flavobacterium			0.354						0.003	1
Erysipelotrichaceae_UCG-009									0.003	1
Kandleria								0.363	0.003	2
unas.Firmicutes						0.325			0.002	1
Frondihabitans						0.301			0.002	1
unas.Yersiniaceae						0.388			0.002	1
unas.Actinomarinales									0.002	1
Gardnerella			0.301						0.002	1
Parvibacter	-0.382	-0.427					0.386		0.001	4
Alkalibaculum				-0.435	0.391		0.348		0.001	3

[Eubacterium]_ruminantium	-0.302								0.001	1
Anaerosporobacter									0.001	1
Massilia									0.001	1
unas.Propionibacteriaceae							0.319		0.001	2
unas.Sphingomonadaceae						0.363			0.0003	1

Figure S5. Beta diversity matrices Bray-Curtis dissimilarity and Weighted UniFrac distance for milk samples taken from the left and right quarter on d30 from all 18 cows. Samples from one cow have the same color. Cow 1 (red) was excluded from further analysis.

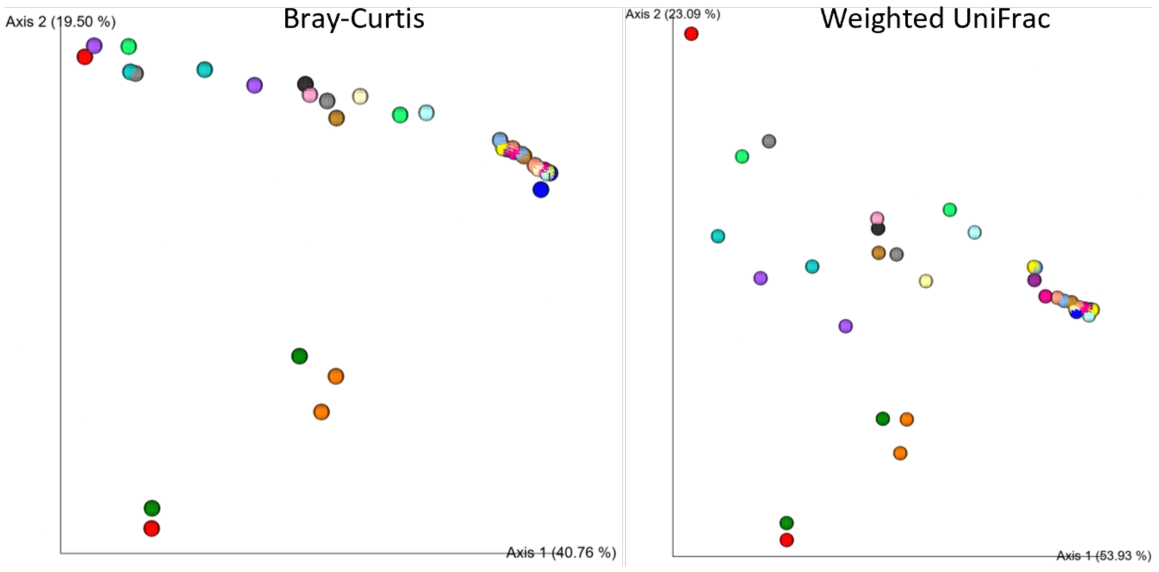

Supplement: Supplementary file 1 — Additional File 1 [file 42523_2025_499_MOESM1_ESM.pdf]
